# RIPK1 inhibition contributes to lysosomal membrane stabilization in ischemic astrocytes via a lysosomal Hsp70.1B-dependent mechanism

**DOI:** 10.1038/s41401-023-01069-8

**Published:** 2023-04-13

**Authors:** Hua-ping Du, Yi Guo, Yong-ming Zhu, De-fei Gao, Bo Lin, Yuan Liu, Yuan Xu, Ali Said, Taous Khan, Li-jun Liu, Jian-jun Zhu, Yong Ni, Hui-ling Zhang

**Affiliations:** 1 Department of Neurology, Suzhou Ninth People’s Hospital, Suzhou Ninth Hospital Affiliated to Soochow University, Soochow University, Suzhou, 215200 China; 2 Jiangsu Key Laboratory of Neuropsychiatric Diseases and College of Pharmaceutical Sciences, Suzhou Key Laboratory of Drug Research for Prevention and Treatment of Hyperlipidemic Diseases, Department of Pharmacology, College of Pharmaceutical Science, Soochow University, Suzhou, 215123 China; 3 Department of Pharmacy, COMSATS University Islamabad, Abbottabad Campus, Islamabad, Pakistan; 4 Emergency Department, The Second Affiliated Hospital of Soochow University, Soochow University, Suzhou, 215004 China; 5 Pain Department, The Second Affiliated Hospital of Soochow University, Soochow University, Suzhou, 215004 China

**Keywords:** ischemic stroke, astrocyte, lysosome, RIPK1, Hsp70.1B, Necrostatin-1

## Abstract

Receptor-interacting protein kinase 1 (RIPK1) contributes to necroptosis. Our previous study showed that pharmacological or genetic inhibition of RIPK1 protects against ischemic stroke-induced astrocyte injury. In this study, we investigated the molecular mechanisms underlying RIPK1-mediated astrocyte injury in vitro and in vivo. Primary cultured astrocytes were transfected with lentiviruses and then subjected to oxygen and glucose deprivation (OGD). In a rat model of permanent middle cerebral artery occlusion (pMCAO), lentiviruses carrying shRNA targeting RIPK1 or shRNA targeting heat shock protein 70.1B (Hsp70.1B) were injected into the lateral ventricles 5 days before pMCAO was established. We showed that *RIPK1* knockdown protected against OGD-induced astrocyte damage, blocked the OGD-mediated increase in lysosomal membrane permeability in astrocytes, and inhibited the pMCAO-induced increase in astrocyte lysosome numbers in the ischemic cerebral cortex; these results suggested that RIPK1 contributed to the lysosomal injury in ischemic astrocytes. We revealed that *RIPK1* knockdown upregulated the protein levels of Hsp70.1B and increased the colocalization of Lamp1 and Hsp70.1B in ischemic astrocytes. *Hsp70.1B* knockdown exacerbated pMCAO-induced brain injury, decreased lysosomal membrane integrity and blocked the protective effects of the RIPK1-specific inhibitor necrostatin-1 on lysosomal membranes. On the other hand, *RIPK1* knockdown further exacerbated the pMCAO- or OGD-induced decreases in the levels of Hsp90 and the binding of Hsp90 to heat shock transcription factor-1 (Hsf1) in the cytoplasm, and *RIPK1* knockdown promoted the nuclear translocation of Hsf1 in ischemic astrocytes, resulting in increased Hsp70.1B mRNA expression. These results suggest that inhibition of RIPK1 protects ischemic astrocytes by stabilizing lysosomal membranes via the upregulation of lysosomal Hsp70.1B; the mechanism underlying these effects involves decreased Hsp90 protein levels, increased Hsf1 nuclear translocation and increased Hsp70.1B mRNA expression.

## Introduction

Stroke is the second leading cause of death worldwide; stroke has become the number one cause of death in China, and its prevalence and morbidity rates have significantly increased. Ischemic stroke accounts for approximately 87% of all stroke cases [1]. To date, specific plasminogen activators, such as tissue plasminogen activator (t-PA) and TNK t-PA, are the main thrombolytic agents that have been approved for the treatment of acute ischemic stroke by the United States Food and Drug Administration. Due to the narrow therapeutic window, only a small number of patients receive intravenous thrombolytic therapy in time [2, 3], indicating an urgent need for new therapeutic strategies for the treatment of ischemic stroke.

Astrocytes are the largest cell population in the central nervous system. Astrocytes mediate various processes, including angiogenesis, neurogenesis, synaptogenesis, and axonal remodeling, in response to ischemic stroke [4–8]. Therefore, astrocytes have become an important subject of research in the pathophysiology of cerebral ischemia and other neurological diseases.

Necroptosis is a highly regulated form of necrosis, and it can be activated by extracellular and intracellular stimuli that induce the expression of death receptor ligands [9, 10]. Receptor-interacting protein kinase 1 (RIPK1) is a crucial mediator of necroptosis. Receptor-interacting protein kinase 3 (RIPK3), which is a key downstream mediator of necroptosis, can be recruited by activated RIPK1. These two kinases interact with each other and form the RIPK1–RIPK3 complex (necrosomes) in necroptotic cells. RIPK1 contains three domains: an N-terminal kinase domain, an intermediate domain and a C-terminal death domain. The N-terminal Ser/Thr kinase domain is involved in the regulation of necroptosis [11–14], and this domain can be inhibited by the small molecule inhibitor necrostatin-1 (Nec-1, which is a specific inhibitor of RIPK1) [12].

Our previous studies demonstrated that RIPK1/RIPK3 activation mediates astrocyte necroptosis in rat models of permanent middle cerebral occlusion (pMCAO) and in astrocytes subjected to oxygen and glucose deprivation (OGD)-induced injury, and the underlying mechanisms include RIPK1 inducing lysosomal membrane permeability (LMP) and cathepsin B release from lysosomes into the cytoplasm in ischemic astrocytes [15]. However, it is unclear how RIPK1 increases LMP.

Lysosomes mainly maintain cell homeostasis and intracellular organelle recycling; lysosomes are the main site of constituent cell protein degradation and amino acid recycling, and they contain a large number of acidic hydrolases. Cathepsins are a representative group of lysosomal hydrolases, and they are classified into three subgroups: cysteine (cathepsins B, C, F, H, K, L, N, O, S, T, U, W and X), aspartyl (cathepsins D and E) and serine (cathepsins A and G) cathepsins. Cathepsins B, L, and D are mainly expressed in astrocytes [16, 17]. The lysosomal membrane is a physical barrier that essentially prevents hydrolytic enzymes from digesting cellular proteins. The destabilization of lysosomal membranes not only influences normal activities but also affects cell vitality. Lysosomal rupture in astrocytes occurs during cerebral ischemia [18]. However, the molecular mechanisms underlying lysosomal membrane rupture are still poorly understood.

Heat shock protein 70.1 (Hsp70.1) is a major protein of the human Hsp70 family. Hsp70.1 mainly functions as a chaperone that allows cells to cope with harmful aggregates of denatured proteins during and after exposure to various insults, such as heat, ischemia, and other oxidative stresses [19, 20]. In addition to the role of chaperones in protecting cells from the effects of damaged proteins, Hsp70.1, which is localized to the lysosomal membrane, is known to stabilize lysosomal membranes by recycling damaged proteins and protecting cells from oxidative stresses [21–23]. In monkey hippocampal CA1 neurons subjected to ischemia–reperfusion insult, Sahara et al. demonstrated that Hsp70.1 is carbonylated by artificial oxidative stressors, such as hydroxynonenal or hydrogen peroxide, and carbonylated Hsp70.1 in CA1 tissues is much more vulnerable to calpain cleavage [24]. Therefore, Hsp70.1 is a modulator of lysosomal rupture/permeabilization after ischemia–reperfusion injury.

Heat shock protein 90 (Hsp90) and heat shock transcription factor-1 (Hsf1) act as coregulators that promote the expression of Hsp70 family proteins [25]. Normally, Hsp90 interacts with Hsf1 to form a chaperone complex in the cytoplasm, and its binding to Hsp90 inhibits the nuclear translation of Hsf1. During ischemia, Hsf1 can be released from the cytoplasmic Hsp90 complex, and Hsf1 forms its own trimer, which has DNA binding ability, enters the nucleus, binds to DNA and promotes the expression of Hsp70 protein family members.

Hsp90 participates in cellular emergency responses, is a molecular chaperone protein in cells, and regulates a variety of client proteins. RIPK1 and RIPK3 are client proteins of Hsp90. A large number of studies have shown that Hsp90 plays an important role in the stability and normal function of RIPK1 [26–28]. Activated molecular chaperone complexes composed of Hsp90 and CDC37 regulate the formation of RIPK1/RIPK3/MLKL complexes, therefore, regulating necroptosis in cells, and Hsp90 inhibitors can inhibit the occurrence of necroptosis [29, 30]. In contrast, several recent studies have shown the effects of necroptotic kinases on Hsp90 levels. These studies showed that inhibition of RIPK1 activation by Nec-1 decreases the Hsp90 protein level and the interaction of Hsp90 and RIPK1/RIPK3/MLKL during TNF-α-, Smac mimetic- and ZVAD-induced human pulmonary artery endothelial cell necroptosis [30, 31]; these results suggest that the interaction of RIPK1 and Hsp90 may also play a key role in the stability of Hsp90. Therefore, RIPK1 may be involved in regulating the level of Hsp90.

In this study, we showed that inhibition of RIPK1 stabilizes lysosomal membranes via the upregulation of lysosomal Hsp70.1B; the underlying molecular mechanism is associated with the inhibition of RIPK1, which decreases the levels of Hsp90 and its interaction with Hsf1, in turn promoting the nuclear translocation of Hsf1 and increasing the expression of its target gene Hsp70.1B.

## Materials and methods

### Animals

Male Sprague‒Dawley (SD) rats (weight: 280–320 g, fasting blood glucose: 4~5.5 mmol/L) were purchased from SLAC Company (Shanghai, China). All the animal procedures and protocols used in this study were approved by the Soochow University Animal Care and Use Committee (use license: SYXK-2016-0050; production license: SYXK-2017-0006). All the animals were fed a standard diet and were maintained under 12-h cycles of light and darkness. All the procedures were designed to minimize both animal suffering and the number of animals used.

### Knockdown of *RIPK1* and *Hsp70.1B*

Lentivirus transfection was performed as previously described [15]. Briefly, lentiviruses carrying short hairpin RNA targeting rat *RIPK1* (shRNA RIPK1) or *Hsp70.1B* (shRNA Hsp70.1B) and lentiviruses carrying control scrambled shRNA (scr shRNA) were produced by GeneChem (GeneChem Co., Ltd., China). The target sequence of shRNA RIPK1 was 5′-GCAGTTCTTGGTCTGCATA-3′, the target sequence of shRNA Hsp70.1B was 5′-GGTCCCTGAGTAAATTG-3′, and the target sequence of scr shRNA was 5′-TTCTCCGAACGTGTCACGT-3′.

### Permanent middle cerebral artery occlusion (pMCAO) rat model

#### Rat model of pMCAO

pMCAO was established as previously described [15, 32]. The rats were randomly assigned to groups by using the online tool Quickcalcs (http://www.graphpad.com/quickcalcs/). Briefly, after anesthetic treatment of the rats, a rubber silicon-coated monofilament suture (nylon monofilament size: 4-0, length: 28 mm; diameter with coating: 0.36 ± 0.02 mm; coating length: 5 mm) was inserted into the right internal carotid artery via the common carotid artery and was advanced until the tip of the monofilament occluded the origin of the right middle cerebral artery. The study inclusion criterion of diminished blood flow (>80%) was assessed with laser-Doppler flowmetry (moorVMS, UK). The body temperature of the rats was maintained in the range of 37.0 ± 0.5 °C with a heating pad throughout the entire surgical procedure. Sham-operated rats underwent the same procedures, except that a small incision was not made and a suture was not inserted into the artery.

**Fig. 1:**
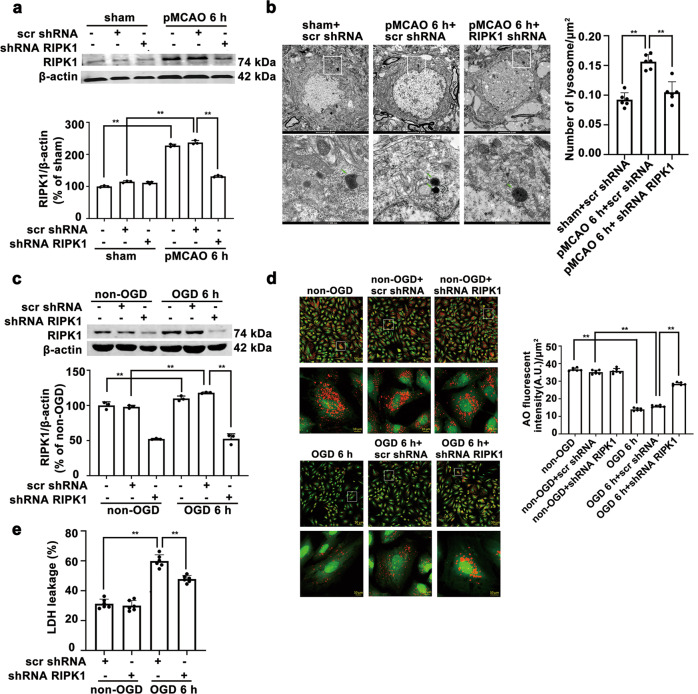
*RIPK1* knockdown increases lysosomal membrane stability and reduces ischemic astrocyte injury. **a**, **b** *RIPK1* knockdown prevents the activation of lysosomes in ischemic astrocytes of the cerebral cortex in pMCAO model rats. **a** Western blotting results show that *RIPK1* was successfully knocked down in the rat cerebral cortex. Columns represent quantitative analysis of immunoblots. Means ± SDs, *n* = 3. **P < 0.01. **b** Representative transmission electron microscopy images show that *RIPK1* knockdown reduces the number of lysosomes in astrocytes of the cerebral cortex in pMCAO model rats. N nucleus. The green arrow indicates lysosomes. Scale bars indicate 2 μm. Magnified images are cropped sections from the areas indicated with white borders in the images, and scale bars indicate 500 nm. Means ± SDs, *n* = 6. **P < 0.01. **c**–**e** *RIPK1* knockdown blo**c**ks OGD-induced lysosomal membrane damage and reduces ischemic astrocyte injury. **c** Western blotting results show that *RIPK1* was successfully knocked down in astrocytes. Columns represent quantitative analysis of immunoblots. Means±SDs, *n* = 3. **P < 0.01. **d** Representative photomicrographs of AO staining. Cells were subjected to OGD for 6 h and then incubated with AO (5 μg/mL) for 15 min. Scale bars indicate 50 μm. Magnified images are cropped sections from the areas indicated with white borders, and scale bars indicate 10 μm. Quantitative analysis of the red fluorescence intensity of AO staining was conducted. Means ± SDs, *n* = 6. **P < 0.01. **e** *RIPK1* knockdown reduces OGD-induced LDH release by astrocytes. Means ± SDs, *n* = 6. **P < 0.01. Statistical analysis was performed with one-way ANOVA followed by a *post hoc* Tukey test.

*RIPK1* or *Hsp70.1B* knockdown (shRNA RIPK1 or shRNA Hsp70.1B) or control (scr shRNA) lentiviruses were injected intracerebroventricularly (i.c.v) stereotaxically at the coordinates of 1.5 mm posterior to the bregma, 1.5 mm lateral from the midline, and 4.0 mm depth to the cortical surface above the lateral ventricles 5 days before pMCAO was established. The transfection efficiency was greater than 80%. Western blotting analysis confirmed that *RIPK1* (Fig. 1a) and *Hsp70.1B* (Fig. 4a) were successfully silenced in the rat brain. For Nec-1 treatment, 24 nmol Nec-1 (N9037, Sigma-Aldrich, USA) or vehicle was intracerebroventricularly administered upon occlusion of the middle cerebral artery.

To observe cerebral infarction, the forebrains were divided into five coronal sections (3 mm) using a rat brain matrix (Harvard apparatus); then, the sections were subsequently incubated in 2% 2,3,5-triphenyltetrazolium chloride (TTC, Sigma-Aldrich, USA) (Xu et al., 2014) at 37 °C for 30 min followed by fixation in 4% paraformaldehyde in PBS (pH 7.4). The infarction volume was calculated with ImageJ software and expressed as the percentage of the ipsilateral volume.

#### Assessment of neurological deficits

Neurological deficits scores, grip strength tests and limb-use asymmetry tests were used to assess neurological deficits as previously described [33]. Behavioral tests were performed before pMCAO and at 6 h after pMCAO by an investigator who was blinded to the experimental groups.

##### Neurological deficit scores

A grading scale of 0–5 was used to assess the neurological function deficit, and the scale is briefly described as follows: 0, no observable neurological function deficit; 1, failure to fully extend contralateral forepaw; 2, circling of the contralateral forepaw to the contralateral side; 3, falling to the contralateral side at rest; 4, no spontaneous motor activity and a depressed level of consciousness; and 5, died after recovery from anesthesia.

##### Grip strength test

Forelimb grip strength was evaluated using a rat grip strength meter (YLS-13A, Jinan Yi Yan Technology Development Company Limited, China). We grabbed the rat tails and lifted them up so that they grasped the pull bar with both forepaws. The peak force was recorded as the maximum grip strength (strength units recorded as grams). The data are presented as averages from 10 tests for each rat.

##### Cylinder test (limb-use asymmetry test)

Rats were placed into a Plexiglas cylinder (HOOFAN, China), and the number of times the forelimbs contacted the wall during the rat’s vertical movements along the wall of the cylinder was analyzed. A total of 20 rat movements were recorded, and the final cylinder score was calculated as follows: (Right forelimb movement – Left forelimb movement) / (Right forelimb movement + Left forelimb movement + Both movements).

### Transmission electron microscopy

Transmission electron microscopy was used to analyze the protective effect of *RIPK1* knockdown on the ischemic cortex after pMCAO as previously described [34]. Six hours after pMCAO, 1 cubic millimeter of brain fragments were harvested from the ischemic core of the mouse cortex and processed as follows. The samples were postfixed in 1% osmium tetroxide in 0.1 mol/L phosphate buffer (pH 7.4) for 1 h, dehydrated in a graded ethanol series, and flat embedded in epoxy resin. Ultrathin sections (40- to 60-nm-thick) were cut with a Reichert ultramicrotome and placed on grids (200 mesh), stained with uranyl acetate and lead citrate, and then observed under a Philips CM-120 electron microscope.

### Primary cortical astrocyte culture, oxygen-glucose deprivation (OGD), and cell death assessment

#### Primary cortical astrocyte culture

Primary cortical astrocyte cultures were prepared from 24 h postnatal SD rats as previously described [16]. Briefly, the cerebral cortices were digested with 2.5% trypsin for 10 min at 37 °C and filtered through a sterile 40 μm nylon cell strainer. The cells were suspended in Dulbecco’s modified Eagle’s medium (DMEM)/F12 (1:1) (11330, Gibco, USA) supplemented with 10% fetal bovine serum (10099, Gibco, USA) and 1% penicillin–streptomycin (C0222, Beyotime, China) solution. The cells were maintained at 37 °C under >90% humidity in 5% CO_2_. The medium was changed every 2–3 days until the cells reached confluence. The population of cells obtained with this procedure included more than 95% astrocytes, which were positive for glial fibrillary acid protein (GFAP) staining.

#### Oxygen-glucose deprivation (OGD)

The OGD model was established as previously described [16]. Primary cultured astrocytes were rinsed twice with phosphate-buffered saline (PBS, pH 7.4), and then fresh glucose-free DMEM (11966, Gibco, USA) was added. The cells were placed in a sealed chamber (Billups-Rothenberg, USA), and mixed gas containing 95% N_2_ and 5% CO_2_ was introduced into the sealed chamber for approximately 10 min to exclude oxygen. Then, the cells were incubated in the sealed hypoxia chamber at 37 °C (the incubation time depended on the experimental requirements). For treatment with Nec-1, 100 μM Nec-1 was added to the astrocytes upon OGD.

*RIPK1* or *Hsp70.1B* knockdown (shRNA RIPK1 or shRNA Hsp70.1B) or control (scr shRNA) lentiviruses were diluted with enhanced infection solution and added to the third passage of primary cultured astrocytes (1 × 10^8^ TU/mL, 10 μL). Then, the cells were transfected for 72 h, and the transfection efficiency was greater than 80%. Western blotting analysis confirmed that *RIPK1* (Fig. 1c) and *Hsp70.1B* (Fig. 4b) were successfully silenced in primary cultured astrocytes.

#### Measurement of lactate dehydrogenase (LDH) release

To analyze cell death, lactate dehydrogenase (LDH) release from astrocytes was measured using an LDH assay kit (A020, Nanjing Jiancheng, China) at 450 nm using a multimode microplate reader, Infinite® M1000 PRO (Tecan Trading AG, Switzerland), as previously described [33].

#### Lysosomal stability assay

The LMP of primary cultured astrocytes was evaluated with acridine orange staining (AO, 318337, Sigma-Aldrich, USA) [15, 35]. AO is a lysomotropic metachromatic fluorophore dye that emits red fluorescence in its protonated form in lysosomes. When LMP is increased, AO is released from lysosomes into the cytosol, leading to the cytoplasmic diffusion of green fluorescence and reduced red fluorescence. Cells were subjected to OGD for 6 h and then incubated with 5 μg/mL AO in a complete medium for 15 min at 37 °C. Images were acquired using a confocal laser scanning microscope (LSM 710, Carl Zeiss, Germany).

### Western blotting analysis

Proteins were extracted from ipsilateral cortical tissues or cultured astrocytes and used for Western blotting analysis as previously described [36]. Proteins were separated on 8%–12% SDS–PAGE gels and transferred to nitrocellulose membranes. The membranes were blocked in 5% BSA or 5% nonfat dry milk and then incubated overnight at 4 °C with the primary antibodies (Supplementary Table S1). Then, the membranes were washed and incubated with secondary antibodies (Supplementary Table S2) for 1 h at room temperature and washed. The blots were visualized with an Odyssey scanner (LI-COR), and quantitative densitometric analyses of the bands were performed with ImageJ software.

### Immunohistochemistry and immunofluorescence

Immunohistochemistry was performed as previously described [16]. Brain sections were fixed with 4% paraformaldehyde for 10 min, permeabilized with 0.3% Triton X-100, blocked with 1% BSA for 1 h at room temperature, and incubated with specific primary antibodies (Supplementary Table S1) overnight at 4 °C followed by the corresponding secondary antibodies (Supplementary Table S2) for 1 h at room temperature. Hoechst (1:5000, 33258, Sigma-Aldrich, USA) was used to counterstain the nuclei. The images were captured by a confocal microscope (LSM 710, Carl Zeiss Co. Ltd., Germany). Fluorescence intensity was determined using built-in and custom-written ImageJ plugins and normalized to the background. The results are expressed as the mean fluorescence intensity (fluorescence intensity per unit area, A.U./μm^2^). FIJI ImageJ software colocalization finder was used to analyze the colocalization of double-stained images. Manders’ overlap coefficient indicates the actual overlap between color channels, which represents the true level of colocalization. In the range of values from 0 to 1, the results are as follows: 1 indicates complete overlap, and 0 indicates no overlap. Manders’ overlap coefficients are suitable when the fluorescence of one antigen is stronger than that of another antigen.

For immunofluorescence, the cultured cells were subjected to OGD for 6 h, rinsed with PBS, fixed in 4% paraformaldehyde, permeabilized with 0.1% Triton X-100, blocked with 1% BSA, and then incubated overnight at 4 °C with specific primary antibodies (Supplementary Table S1). Then, the cells were subsequently incubated with the corresponding secondary antibodies (Supplementary Table S2) at room temperature for 1 h. After being washed with PBS, the cells were incubated in Hoechst (1:5000, 33258, Sigma-Aldrich, USA) solution for 10 min to counterstain the nuclei. Images were captured with a confocal microscope (LSM 710; Carl Zeiss Co. Ltd., Oberkochen, Germany) [16].

### Nuclear–cytoplasmic fractionation

Nuclear–cytoplasmic fractionation was conducted using the NE-PER Nuclear and Cytoplasmic Extraction Reagents kit (Thermo Fisher Scientific, USA) according to the manufacturer’s protocol. Briefly, treated cells were washed twice with cold PBS and centrifuged at 500 × g for 3 min. The cell pellets were suspended in 200 mL of cytoplasmic extraction reagent I by vortexing. The suspensions were incubated on ice for 10 min. Then, 11 mL cytoplasmic extraction reagent II was added, and the samples were vortexed for 5 s, incubated on ice for 1 min and centrifuged for 5 min at 16,000 ×*g*. The supernatant fractions (cytoplasmic extracts) were transferred to prechilled tubes. The insoluble pellet fractions, which contained crude nuclei, were resuspended in 100 mL of nuclear extraction reagent by vortexing for 15 s, incubated on ice for 10 min, and then centrifuged for 10 min at 16,000 ×*g*. The resulting supernatant fractions, which constituted nuclear extracts, were used for subsequent experiments.

### Coimmunoprecipitation

Lysates were incubated with an anti-Hsf1 primary antibody (1:500, ab2923, Abcam, UK) overnight at 4 °C with rotation to form an immunocomplex. Normal IgG was used in parallel as an isotype control for the anti-Hsf1 antibody. Protein A/G magnetic beads (B23201, Bimake, China) were prewashed twice with cell lysis buffer. A magnetic separation rack was used to separate magnetic beads from a liquid buffer. Subsequently, the immunocomplexes were incubated with the prewashed magnetic beads for 30 min at room temperature with rotation. The bead-bound immunocomplexes were washed five times with lysis buffer, resuspended in loading buffer and heated at 95 °C for 5 min to release the beads. The free beads were removed from the samples using a magnetic separation rack, and then, the samples were subjected to Western blotting.

### Statistical analysis

The data are presented as the means ± SDs. Statistical analysis for multiple comparisons was performed with one-way analysis of variance (ANOVA) followed by a *post hoc* Tukey’s test, and the difference between the two groups was evaluated by unpaired Student’s *t* test. Nonparametric data, such as neurological deficit scores, were evaluated by the Mann‒Whitney U test. *P* < 0.05 was considered statistically significant.

## Results

### *RIPK1* knockdown increases lysosomal membrane stability and reduces ischemic astrocyte injury

Knockdown of *RIPK1* in the rat cerebral cortex and primary cultured astrocytes was verified (Fig. 1a, c). Transmission electron microscopy results showed that after pMCAO insult, astrocyte lysosomes were darkly stained, and the number of lysosomes was increased. *RIPK1* knockdown reduced the number of astrocyte lysosomes in the cerebral ischemic area and improved the color of the lysosomes, suggesting that *RIPK1* knockdown protects the lysosomal structure of astrocytes in the ischemic area (Fig. 1b). In vitro, we measured LMP with an AO staining assay. As shown in Fig. 1d, 6 h of OGD decreased the red fluorescence in astrocytes. In contrast, *RIPK1* knockdown markedly inhibited the OGD-induced reduction in red granular fluorescence of AO staining, indicating that *RIPK1* knockdown decreases LMP during OGD-induced astrocyte injury. Moreover, *RIPK1* knockdown significantly reduced LDH release from OGD-treated astrocytes (Fig. 1e).

### *RIPK1* knockdown or Nec-1 treatment further increases the levels of lysosomal Hsp70.1B in ischemic astrocytes

**Fig. 2:**
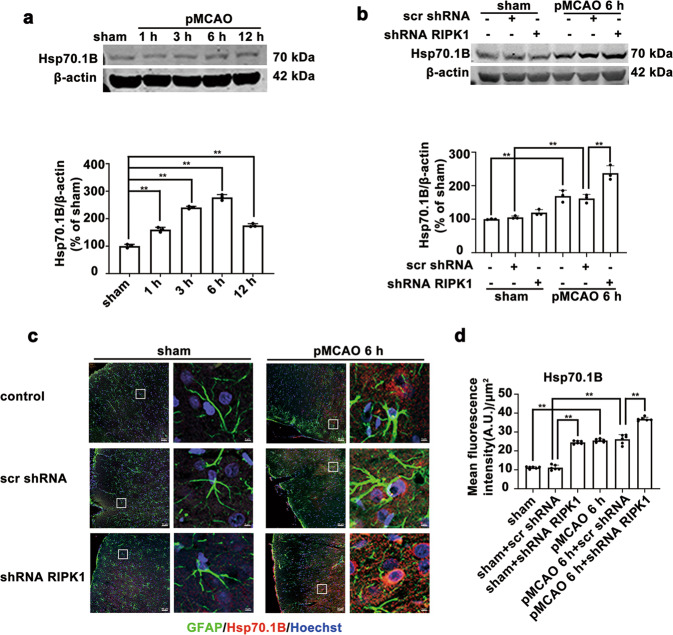
Knockdown of *RIPK1* further increases the Hsp70.1B protein levels in the astrocytes of the ischemic cerebral cortex of rats. **a**, **b** Western blotting results show that the protein levels of Hsp70.1B were increased in the cerebral cortex from 1 to 12 h after pMCAO, and *RIPK1* knockdown further increased the protein levels of Hsp70.1B at 6 h post-pMCAO. Columns represent quantitative analysis of immunoblots. Means ± SDs, *n* = 3. **P < 0.01. **c**, **d** *RIPK1* knockdown increases the Hsp70.1B levels in astrocytes of the ischemic cerebral cortex. **c** Representative photomicrographs of double immunostaining of Hsp70.1B and GFAP in the ischemic cortex (Hsp70.1B: red; GFAP: green; Hoechst: blue). GFAP immunolocalization was visualized with astrocyte markers. Scale bars indicate 50 μm. Magnified images are cropped sections from the areas indicated with white borders, and scale bars indicate 10 μm. **d** Quantitative analysis of Hsp70.1B fluorescence intensity in **c**. Means ± SDs, *n* = 6. **P < 0.01. Statistical analysis was performed with one-way ANOVA followed by a post hoc Tukey test.

Hsp70 plays an important role in the normal physiological function of cells and enhances the tolerance of cells to stress conditions [37], and its expression can be rapidly and substantially induced under hypoxic conditions. Hsp70.1, which is a major protein of the human Hsp70 family, is known to stabilize the lysosomal membrane by recycling damaged proteins and protecting cells from oxidative stresses and ischemic/reperfusion injury. The literature indicates that lysosomal Hsp70.1 expression is increased in monkey hippocampal CA1 neurons after ischemia/reperfusion insult, contributing to lysosomal membrane stabilization and reducing the release of lysosomal cathepsins into the cytosol [21]. Hsp70.1 is expressed as either Hsp70.1A or Hsp70.1B, and these two subtypes share more than 99% similarity in their amino acid sequences. We found that the Hsp70.1B level was increased in the cerebral cortex of rats from 1 to 12 h after pMCAO and peaked at 6 h post-pMCAO (Fig. 2a). Western blotting results (Fig. 2b) showed that *RIPK1* knockdown further upregulated the Hsp70.1B protein level in the ischemic cerebral cortex of rats. Immunofluorescence histochemistry analysis revealed that *RIPK1* knockdown further increased the Hsp70.1B protein level in GFAP-positive astrocytes in the ischemic cerebral cortex of rats (Fig. 2c, d).

**Fig. 3:**
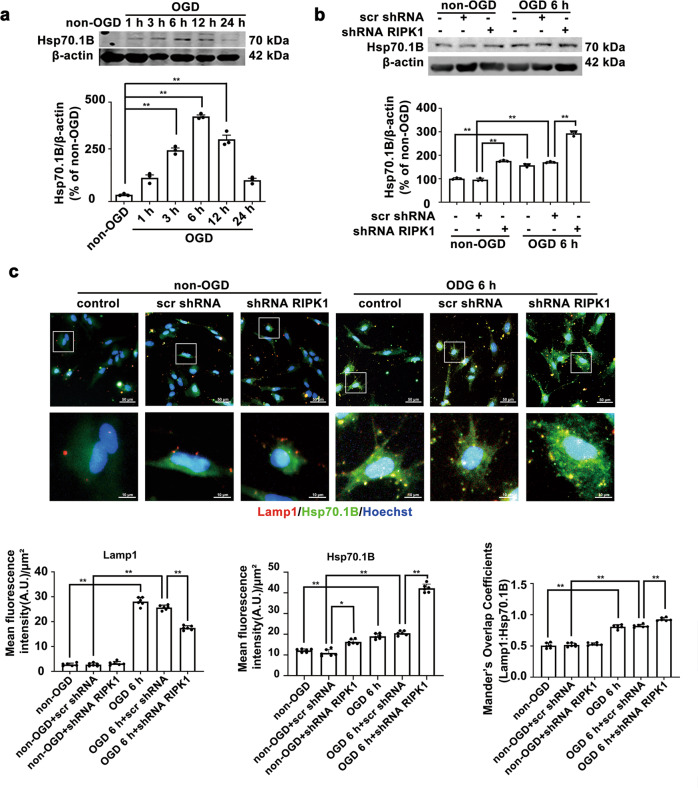
Knockdown of *RIPK1* increases the Hsp70.1B levels in astrocytes with OGD-induced injury. **a**, **b** Western blotting results show that the protein levels of Hsp70.1B were increased in OGD-treated astrocytes from 1 to 12 h (**a**), and *RIPK1* knockdown further increased the protein levels of Hsp70.1B in astrocytes at 6 h post-OGD (**b**). Columns represent quantitative analysis of immunoblots. Means ± SDs, *n* = 3. **P < 0.01. **c** *RIPK1* knockdown increases the Hsp70.1B protein levels in the lysosomes of ischemic astrocytes. Representative photomicrographs of double immunostaining of Lamp1 and Hsp70.1B in astrocytes (Lamp1: red; Hsp70.1B: green; Hoechst: blue). Scale bars indicate 50 μm. Magnified images are cropped sections from the areas indicated with white borders, and scale bars indicate 10 μm. Columns represent quantitative analysis of the Lamp1 and Hsp70.1B fluorescence intensity after immunostaining. Manders’ overlap coefficient demonstrates the colocalization between Hsp70.1B and Lamp1. Means ± SDs, *n* = 6. *P < 0.05, **P < 0.01. Statistical analysis was performed with one-way ANOVA followed by a *post hoc* Tukey test.

In vitro, the protein level of Hsp70.1B increased from 1 to 12 h after OGD and peaked at 6 h post-OGD (Fig. 3a). Western blotting results showed that *RIPK1* knockdown further increased the Hsp70.1B protein levels in astrocytes after 6 h of OGD (Fig. 3b). The fluorescence intensity of Hsp70.1B was significantly enhanced in Lamp1-positive astrocytes after 6 h of OGD, and *RIPK1* knockdown further upregulated the Hsp70.1B levels and enhanced the colocalization of Hsp70.1B and Lamp1 in astrocytes (Fig. 3c). These results suggested that knockdown of *RIPK1* increases the Hsp70.1B level at the lysosomal membrane in ischemic astrocytes.

### *Hsp70.1B* knockdown decreases lysosomal membrane integrity and blocks the Nec-1-mediated protection of lysosomal membranes in astrocytes

**Fig. 4:**
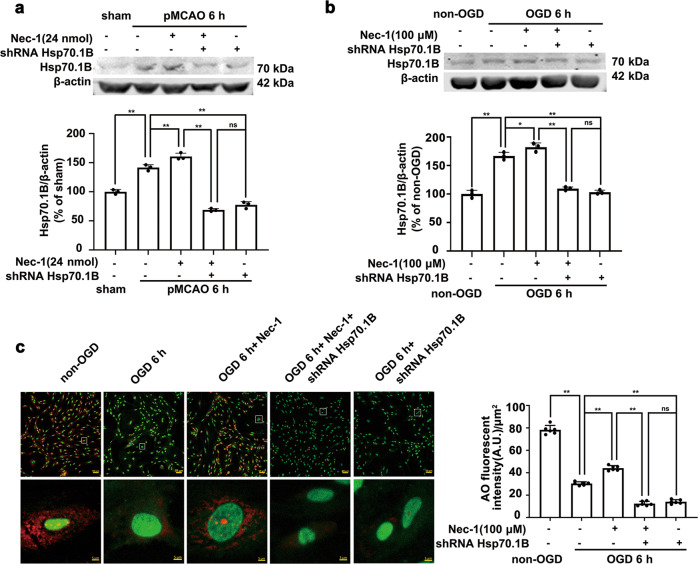
*Hsp70.1B* knockdown decreases lysosomal membrane integrity and blocks Nec-1-mediated protection of lysosomal membranes. **a**, **b** Nec-1-mediated increase in the Hsp70.1B levels is abolished by *Hsp70.1B* knockdown in the ischemic cerebral cortex of rats (**a**) or in OGD-treated astrocytes (**b**). Columns represent quantitative analysis of immunoblots. Means ± SDs, *n* = 3. *P < 0.05, **P < 0.01. **c** Representative photomicrographs of AO staining. Cells were subjected to OGD for 6 h and then incubated with AO (5 μg/mL) for 15 min. Nec-1 (100 μM) was added to the cells 30 min before OGD. Scale bars indicate 50 μm. Magnified images are cropped sections from the areas indicated with white borders, and scale bars indicate 5 μm. Columns represent quantitative analysis of red fluorescence intensity of AO staining. Means ± SDs, *n* = 6. **P < 0.01. Statistical analysis was performed with one-way ANOVA followed by a *post hoc* Tukey test.

To determine whether the lysosomal membrane stabilization mediated by RIPK1 inhibition in ischemic astrocytes depends on lysosomal Hsp70.1B, we knocked down *Hsp70.1B* by using shRNA, and we observed the effects of *Hsp70.1B* knockdown on the Nec-1-mediated increase in Hsp70.1B expression and the protective effect of Hsp70.1B on lysosomal membranes in astrocytes. The results showed that Nec-1, which is a RIPK1-specific inhibitor, increased the Hsp70.1B levels in astrocytes after pMCAO or OGD; in contrast, *Hsp70.1B* knockdown blocked the Nec-1-induced increase in the Hsp70.1B levels (Fig. 4a, b). AO is a lysomotropic metachromatic fluorophore dye that emits red fluorescence in its protonated form in lysosomes. When LMP is increased, AO relocates from lysosomes to the cytosol, leading to cytoplasmic diffusion of green fluorescence and reduced red fluorescence. The red fluorescence of AO staining was significantly reduced at 6 h post-OGD, and Nec-1 treatment recovered the level of red fluorescence of AO staining in OGD-treated astrocytes. In contrast, *Hsp70.1B* knockdown further enhanced the reduction in AO staining in astrocytes subjected to OGD and blocked the restorative effect of Nec-1 on AO red fluorescence. (Fig. 4c). These results indicated that lysosomal membrane stabilization due to RIPK1 inhibition in ischemic astrocytes depends on lysosomal Hsp70.1B.

### *Hsp70.1B* knockdown increases the cerebral infarction volume and exacerbates behavioral disorders

**Fig. 5:**
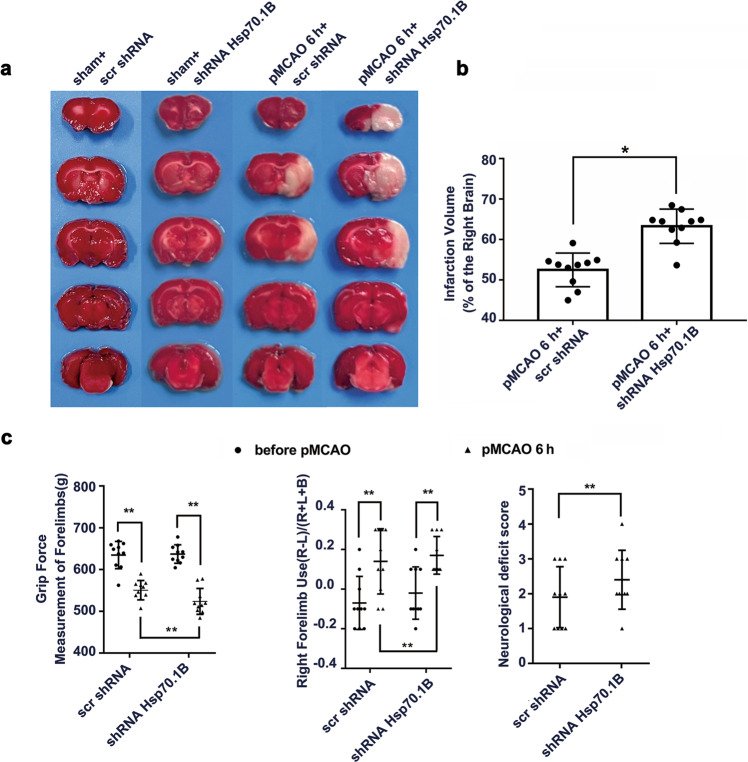
Knockdown of *Hsp70.1B* further exacerbates infarction and behavioral disorders in pMCAO model rats. **a** TTC staining results show that *Hsp70.1B* knockdown increases the cerebral infarction volumes of ischemic rats. **b** Quantitative analysis of infarct volume. Means ± SDs, *n* = 10. *P < 0.05. **c** *Hsp70.1B* knockdown significantly decreases grip force and increases right limb utilization and the neurological deficit score. Means ± SDs, *n* = 8. **P < 0.01. Statistical analysis was carried out with one-way ANOVA followed by a *post hoc* Tukey test for multiple comparisons, Student’s *t* test for comparisons between two groups, and the Mann–Whitney U test for neurological deficit evaluation.

TTC staining showed that *Hsp70.1B* knockdown increased the infarct volume (Fig. 5a, b), decreased grip force, and increased right forelimb utilization and neurological deficit scores in pMCAO model rats (Fig. 5c).

### *RIPK1* knockdown reduces cytoplastic Hsp90 expression and its interaction with Hsf1, increases the nuclear translation of Hsf1, and enhances the mRNA expression of Hsp70.1B

**Fig. 6:**
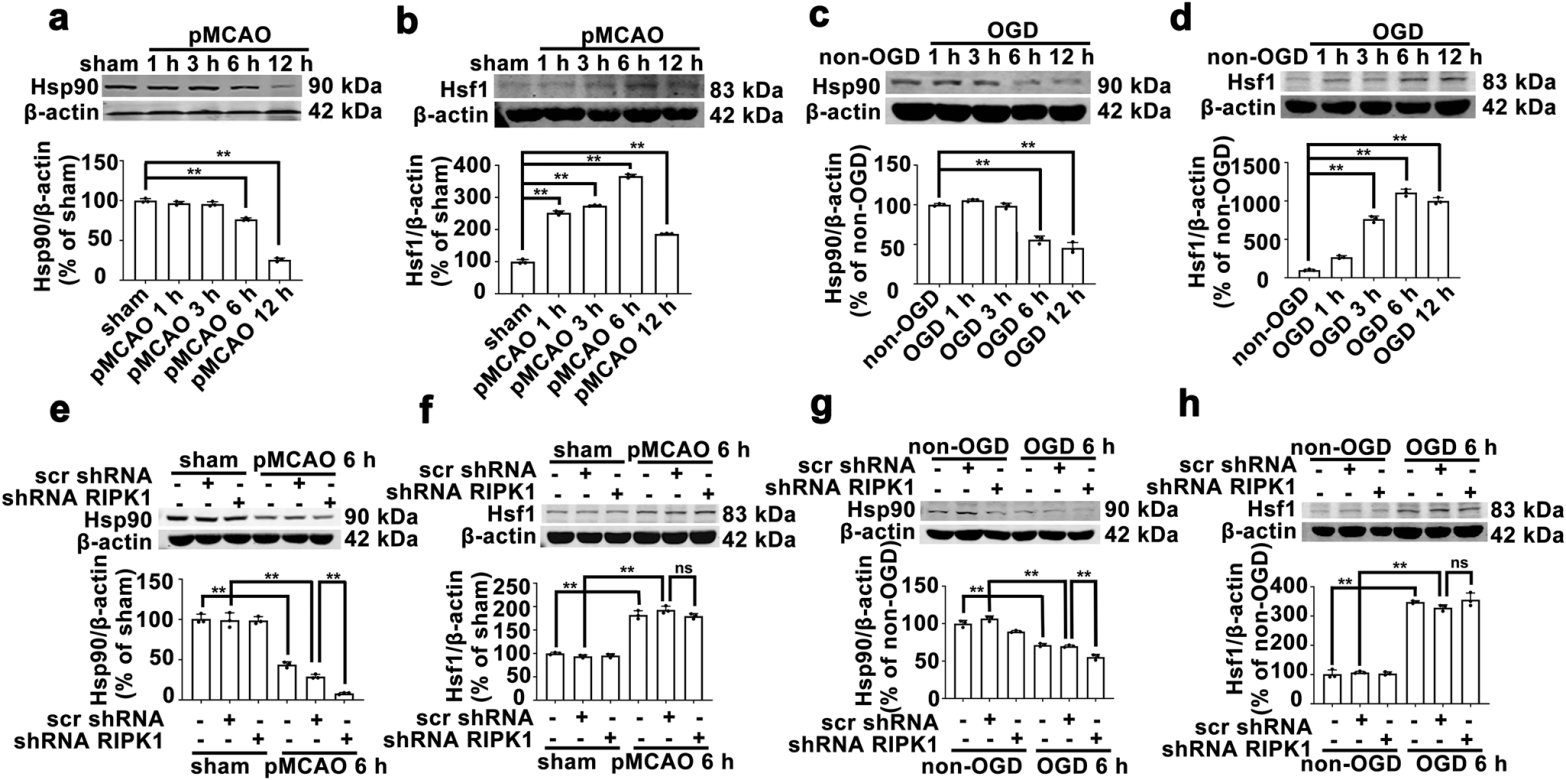
Hsp90 and Hsf1 protein levels in the ischemic cerebral cortex of pMCAO model rats or OGD-treated astrocytes. **a**–**d** Time point changes in the protein levels of Hsp90 and Hsf1 in the ischemic cerebral cortex of pMCAO model rats or OGD-treated astrocytes according to Western blotting analysis. Columns represent quantitative analysis of immunoblots. Means ± SDs, *n* = 3. **P < 0.01. **e**–**h** *RIPK1* knockdown decreases the Hsp90 levels and has no significant effect on the Hsf1 levels in the ischemic cerebral cortex of pMCAO model rats or OGD-treated astrocytes according to Western blotting analysis. Columns represent quantitative analysis of immunoblots. Means ± SDs, *n* = 3. **P < 0.01. Statistical analysis was performed with one-way ANOVA followed by a *post hoc* Tukey test.

**Fig. 7:**
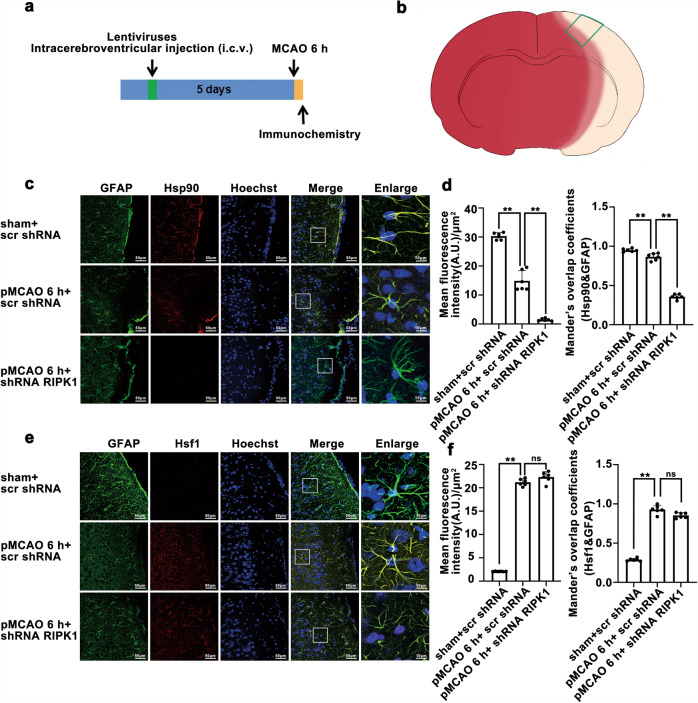
*RIPK1* knockdown reduces the Hsp90 levels but has no significant effect on the total Hsf1 levels in astrocytes from the ischemic cerebral cortex **a** Experimental protocol. **b** Coronal section of brain ideograph showing the image capture location used for immunochemistry of all groups as indicated by the green border area. The white region indicates the infarction region, the red region indicates the non-infarction region, and the pink region indicates the peri-infarction region. **c**, **d** *RIPK1* knockdown further reduced the protein levels of Hsp90 in the cytoplasm of astrocytes from the ischemic cerebral cortex. Representative photomicrographs of double immunostaining for GFAP and Hsp90 in ischemic cerebral cortex (Hsp90: red; GFAP: green; Hoechst: blue). Scale bars indicate 50 μm. Magnified images are cropped sections from the areas indicated with white borders, and scale bars indicate 10 μm. Quantitative analysis of Hsp90 fluorescence intensity after immunostaining. Means ± SDs, *n* = 6. **P < 0.01. Manders’ overlap coefficient demonstrates the colocalization between GFAP and Hsp90. Means ± SDs, *n* = 6. **P < 0.01. **e**, **f** *RIPK1* knockdown has no effect on the protein levels of Hsf1 in the ischemic cerebral cortex. Representative photomicrographs of double immunostaining of GFAP and Hsf1 in the ischemic cerebral cortex (Hsf1: red; GFAP: green; Hoechst: blue). Scale bars indicate 10 μm. Quantitative analysis of Hsf1 fluorescence intensity after immunostaining. Means ± SDs, *n* = 6. **P < 0.01. Manders’ overlap coefficient demonstrates the colocalization between GFAP and Hsf1. Means ± SDs, *n* = 6. **P < 0.01. GFAP immunolocalization was visualized with astrocyte markers. Statistical analysis was performed with one-way ANOVA followed by a *post hoc* Tukey test.

**Fig. 8:**
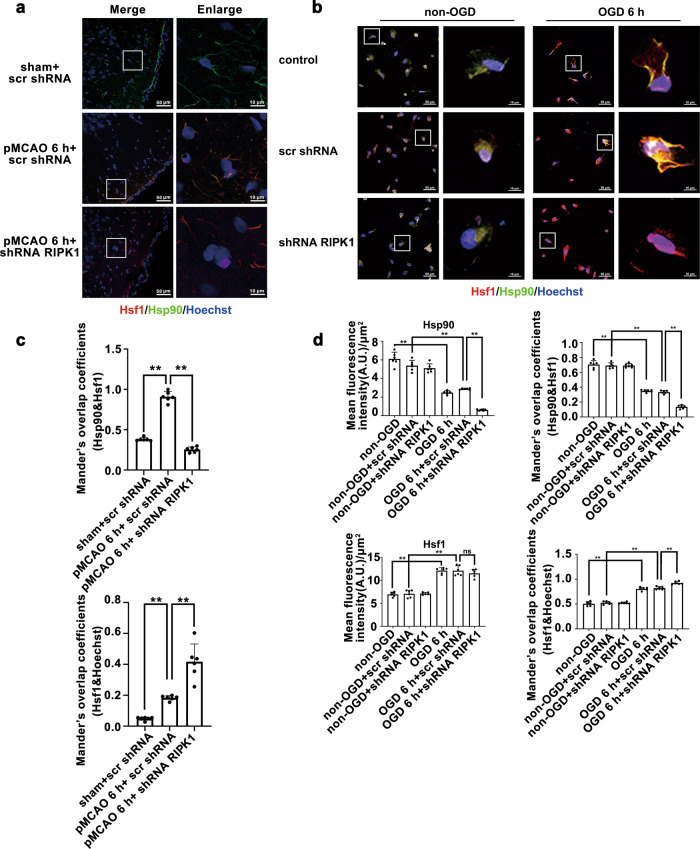
Knockdown of *RIPK1* further decreases the Hsp90 levels in the cytoplasm and increases the translocation of Hsf1 from the cytoplasm to the nucleus in the ischemic cerebral cortex or in OGD-treated astrocytes. **a**, **c** Knockdown of *RIPK1* decreases the Hsp90 levels in the cytoplasm and increases the translocation of Hsf1 from the cytoplasm to the nucleus in the ischemic cerebral cortex. **a** Representative photomicrographs of double immunohistochemistry staining for Hsp90 and Hsf1 in the ischemic cerebral cortex (Hsf1: red; Hp90: green; Hoechst: blue). Scale bars indicate 10 μm. **c** Quantitative analysis of Hsp90/Hsf1 fluorescence intensity in **a**. Manders’ overlap coefficient demonstrates the colocalization between Hsp90 and Hsf1, as well as that between Hsf1 and Hoechst. Means ± SDs, *n* = 6. **P < 0.01. **b**, **d** Knockdown of *RIPK1* decreases the Hsp90 levels in the cytoplasm and increases the translocation of Hsf1 from the cytoplasm to the nucleus in OGD-treated astrocytes. **b** Representative photomicrographs of double immunostaining of Hsp90 and Hsf1 in astrocytes (Hsf1: red; Hsp90: green; Hoechst: blue). Scale bars indicate 50 μm. Magnified images are cropped sections from the areas indicated with white borders. Scale bars indicate 10 μm. **d** Quantitative analysis of Hsp90/Hsf1 fluorescence intensity in **b**. Manders’ overlap coefficient demonstrates the colocalization between Hsp90 and Hsf1 as well as that between Hsf1 and Hoechst. Means ± SDs, *n* = 6. **P < 0.01. Statistical analysis was performed with one-way ANOVA followed by a post hoc Tukey test.

We next tried to answer why RIPK1 inhibition increases lysosomal Hsp70.1B levels in ischemic astrocytes. Hsp90 is a member of the heat shock protein family. Under nonstress conditions, Hsp90 interacts with Hsf1 to form a molecular chaperone complex in the cytoplasm and inhibits the nuclear translocation of Hsf1. Under stress conditions, Hsf1 can be activated in a variety of ways. Hsp90 depletion leads to significant activation of Hsf1 [38, 39]. Activated Hsf1 forms a trimeric complex that translocates to the nucleus and binds to elements in the Hsp70 gene promoter, resulting in a rapid increase in the expression of Hsp70 [40]. Western blotting analysis showed that after pMCAO, the Hsp90 protein level in the rat cerebral cortex was significantly decreased at 6 h and further decreased at 12 h (Fig. 6a). Moreover, the Hsf1 level was increased after pMCAO and peaked at 6 h post-pMCAO (Fig. 6b). In contrast, *RIPK1* knockdown further reduced the Hsp90 level in pMCAO model rats but had no significant effect on the total Hsf1 level (Figs. 6e, f and 7). Immunofluorescence histochemistry analysis further revealed that in the cerebral cortex of sham model rats, Hsf1 and Hsp90 were mainly distributed in the cytoplasm of GFAP-positive astrocytes and exhibited strong spatial colocalization (Fig. 7). In GFAP-positive astrocytes in the ischemic cerebral cortex at 6 h post-pMCAO, the fluorescence intensity of Hsp90 was significantly reduced, while the Hsf1 fluorescence intensity was increased, and Hsf1 had translocated from the cytoplasm to the nucleus. *RIPK1* knockdown further decreased the fluorescence intensity of Hsp90 in the cytoplasm and promoted the nuclear translocation of Hsf1 in pMCAO model rats (Fig. 8a, c).

Similarly, in OGD-treated astrocytes, the Hsp90 level was decreased at 6–12 h post-OGD (Fig. 6c); simultaneously, the Hsf1 level was increased at 3–12 h after OGD and peaked at 6 h (Fig. 6d). In contrast, *RIPK1* knockdown further decreased the Hsp90 levels in OGD-treated astrocytes (Fig. 6g) and had no significant effect on the Hsf1 levels (Fig. 6h). The immunofluorescence staining results revealed that Hsp90 and Hsf1 were distributed in the cytoplasm and colocalized in non-OGD-treated astrocytes (Fig. 8b, d). However, in OGD-treated astrocytes, the fluorescence intensity of Hsp90 in the cytoplasm was significantly decreased, and the fluorescence intensity of Hsf1 in the nucleus was increased. *RIPK1* knockdown further reduced the fluorescence of Hsp90 in the cytoplasm and enhanced the nuclear translocation of Hsf1 in astrocytes treated with OGD.

**Fig. 9:**
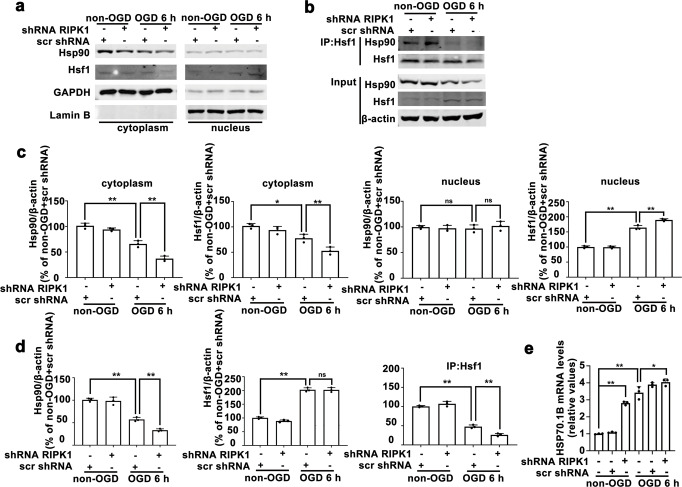
Knockdown of *RIPK1* inhibits the interaction between Hsf1 and Hsp90. **a**, **c** *RIPK1* knockdown decreases the Hsp90 level in the cytoplasm and increases the Hsf1 level in the nucleus in OGD-treated astrocytes, as shown by Western blotting analysis. Columns represent quantitative analysis of immunoblots. GAPDH or Lamin B was used as a loading control in the cytoplasm or nucleus, respectively. Means ± SDs, *n* = 3. *P < 0.05, **P < 0.01. **b**, **d** *RIPK1* knockdown decreases the interaction between Hsf1 and Hsp90 in OGD-treated cells. Cell lysates were subjected to immunoprecipitation with an Hsf1 antibody, and the level of Hsp90 in the immune complex was determined by Western blotting. Columns represent quantitative analysis of immunoblots. Levels of β-actin protein were used as the loading control. Means ± SDs, *n* = 3. **P < 0.01. **e** qPCR results show that *RIPK1* knockdown upregulates Hsp70.1B mRNA expression in OGD-treated astrocytes. Means ± SDs, *n* = 3. *P < 0.05, **P < 0.01. Statistical analysis was performed with one-way ANOVA followed by a *post hoc* Tukey test.

To further investigate the intracellular distribution of Hsp90 and Hsf1, we isolated the cytoplasmic and nuclear fractions of astrocytes after OGD treatment. In OGD-treated astrocytes, the levels of Hsp90 in the cytoplasm were dramatically decreased, and there were no significant changes in the nuclear levels. The Hsf1 levels in the cytoplasmic fraction were markedly decreased, while the levels in the nuclear fraction were significantly increased. *RIPK1* knockdown further enhanced the reduction in the cytoplasmic Hsp90 level and promoted Hsf1 translocation from the cytoplasm to the nucleus in OGD-treated astrocytes (Fig. 9a, c). The results of coimmunoprecipitation showed that Hsf1 interacted with Hsp90 to form a complex in the cytoplasm in non-OGD-treated astrocytes, the interaction between Hsp90 and Hsf1 in astrocytes was reduced after OGD treatment, and *RIPK1* knockdown further decreased the interaction between Hsp90 and Hsf1 (Fig. 9b, d). Next, we verified the effect of RIPK1 knockdown on the mRNA levels of the target gene of Hsf1, namely, Hsp70.1B. Quantitative real-time PCR results demonstrated that OGD significantly upregulated Hsp70.1B mRNA expression in astrocytes, while *RIPK1* knockdown not only increased Hsp70.1B mRNA expression in non-OGD-treated astrocytes but also further increased Hsp70.1B mRNA expression in OGD-treated astrocytes (Fig. 9e). Therefore, these results indicated that *RIPK1* knockdown reduces the levels of Hsp90 and its binding to Hsf1, increasing the translocation of Hsf1 from the cytoplasm to the nucleus and upregulating the mRNA and protein levels of Hsp70.1B.

**Fig. 10:**
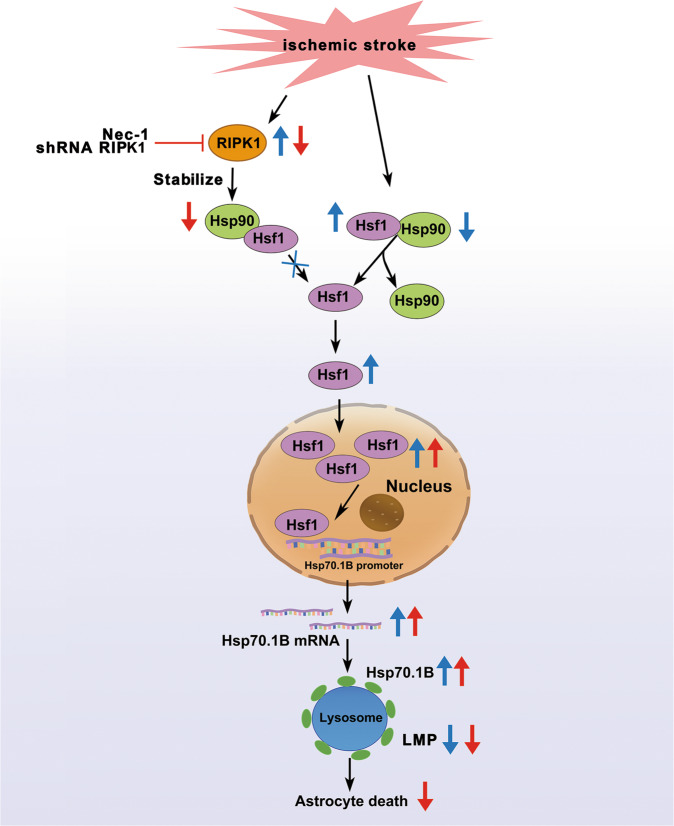
A summary of the findings of the current study. Under normal conditions, Hsp90 interacts with Hsf1 to form a chaperone complex in the cytoplasm, and binding to Hsp90 inhibits the nuclear translation of Hsf1. Hsf1 can be released from the Hsp90 complex in the cytoplasm during ischemic stroke due to the ischemia-mediated decreases in the Hsp90 levels, and Hsf1 forms its own trimeric complex that has DNA binding ability, translocates the nucleus, binds to DNA and promotes the expression of Hsp70 protein family members, which act as protecting protein on the membranes of lysosomes. Genetic inhibition of RIPK1 with shRNA RIPK1 or pharmacological inhibition of RIPK1 with Nec-1 exacerbates ischemic stroke-induced decreases in the levels of Hsp90 and its interaction with Hsf1, which in turn promotes the nuclear translocation of Hsf1 and increases the expression of its target gene *Hsp70.1B*, leading to lysosomal membrane stabilization and astrocytic cell protection.

## Discussion

Our previous study and others have demonstrated that necroptosis, which is a regulated form of necrosis that is mediated by RIPK1 and RIPK3, is induced after ischemic brain injury and is thought to contribute to neuronal and glial cell death [15, 41–44], and this form of cell death may be associated with RIPK1-mediated activation of the autophagic-lysosomal pathway [15, 45]. However, how RIPK1 regulates lysosomal membrane instability in nerve cells under ischemic stroke and other disease conditions is still unknown. Here, we show that genetic or pharmacological inhibition of RIPK1 by gene knockdown or by Nec-1 treatment suppresses pMCAO- or OGD-induced lysosomal membrane destabilization through the upregulation of Hsp70.1B at lysosomal membranes in astrocytes. Furthermore, the mechanism underlying the RIPK1 inhibition-mediated increase in the lysosomal Hsp70.1B levels is related to the effects of *RIPK1* knockdown on enhancing the pMCAO- or OGD-induced decrease in the cytoplasmic levels of Hsp90 and its interaction with Hsf1, which in turn promotes the nuclear translocation of Hsf1 from the cytoplasm and increases Hsp70.1B mRNA transcription.

### Inhibition of RIPK1 protects lysosomal membranes of ischemic astrocytes in an Hsp70.1B-dependent manner

Increasing evidence indicates that lysosomal membrane damage, such as LMP that is associated with the subsequent release of hydrolytic enzymes into the cytosol, occurs during the process of necroptosis [15, 16, 34, 35, 46, 47]. Our previous study and others demonstrated that a variety of cell death stimuli, including ischemia/hypoxia, can increase LMP, resulting in cathepsin release from the lysosomal lumen into the cytoplasm, thus inducing caspase-dependent or caspase-independent cell death [15, 35, 48–52]. Vanden Berghe et al. [53]. showed that lysosomal damage is a late event in TNF-induced necroptosis, whereas inhibition of lysosomal acidification decreases the level of TNF-induced necroptosis [54]. The mechanisms involved in the RIPK1/RIPK3/MLKL cascade activation-mediated LMP increase include the following: (1) Phosphorylated MLKL translocates to the plasma membrane, leading to Ca^2+^ influx. ER stress, as a result of RIPK3 and MLKL phosphorylation, causes Ca^2+^ dysregulation. Increased Ca^2+^ concentrations activate Ca^2+^-dependent enzymes in the cytosol, particularly calpains and cPLA2, which can then increase LMP. (2) Mitochondria- and NOX-dependent ROS induce increased LMP. The generation of ROS relies on necrosomal RIPK3, which in turn facilitates RIPK1 autophosphorylation. Subsequently, disrupted lysosomes release acidic hydrolases (especially cathepsins B and D) into the cytoplasm, resulting in plasma membrane permeability and eventual necroptosis. (3) Lysosomes are essential for the posttranslational regulation of RIPK1 and RIPK3, which guarantees the occurrence of necroptosis. Our previous studies revealed the contribution of RIPK1 to ischemic stroke-induced neuronal and astrocytic cell necroptosis as well as its mechanisms, which are associated with autophagic-lysosomal pathway activation by RIPK1 in vivo and in vitro. These results revealed that during the process of cellular necroptosis, the RIPK1/RIPK3/MLKL-lysosomal signaling pathway plays a crucial role, but the exact mechanism by which RIPK1/RIPK3/MLKL activation is associated with lysosomal membrane damage during necroptosis is still unclear.

The current study revealed for the first time that inhibition of RIPK1 protects the lysosomal membranes of ischemic astrocytes in an Hsp70.1B-dependent manner. Upregulated Hsp70.1 at the lysosomal membrane of hippocampal CA1 neurons is known to stabilize lysosomal membranes after ischemia–reperfusion injury in primates [24]. Both Hsp70.1A and Hsp70.1B are subtypes of Hsp70.1, and they are more than 99% identical, sharing all but two of their 641 amino acids. Basal HSPA1A and HSPA1B mRNA expressions slightly differ in most tissues, with somewhat higher HSPA1A expression in most tissues and cell types. However, the change in the HSPA1B mRNA levels is more obvious than that of the HSPA1A mRNA levels during human traumatic brain injury [55]. In this study, we found that *RIPK1* knockdown protected astrocytes against OGD-induced damage, inhibited the pMCAO-induced increase in the number of astrocyte lysosomes in the ischemic cerebral cortex, and blocked the OGD-mediated increase in LMP in astrocytes; these results suggested that RIPK1 contributes to lysosomal injury in ischemic astrocytes. Further study showed that RIPK1 knockdown upregulated the protein levels of Hsp70.1B and increased the colocalization of Lamp1 and Hsp70.1B in ischemic astrocytes. Furthermore, *Hsp70.1B* knockdown exacerbated infarction volume and behavioral deficits, decreased lysosomal membrane integrity and blocked RIPK1 inhibitor necrostatin-1-mediated protection of lysosomal membranes. These results suggest that inhibition of RIPK1 contributes to protecting lysosomal membranes in ischemic astrocytes in an Hsp70.1B-dependent manner. The compensatory effects of Hsp70.1A may not completely replace the role of Hsp70.1B in the RIPK1-mediated increase in LMP.

### The mechanisms underlying the inhibition of RIPK1-mediated lysosomal Hsp70.1B upregulation

What is the mechanism by which RIPK1 inhibition increases the Hsp70.1B levels at the lysosomal membrane? Under nonstress conditions, Hsp90 interacts with Hsf1 to form a molecular chaperone complex in the cytoplasm and inhibits the nuclear translocation of Hsf1. Under stress conditions, Hsf1 can be activated in a variety of ways. Hsp90 depletion leads to significant activation of Hsf1 [38, 39]. Activated Hsf1 forms a trimeric complex that translocates to the nucleus and binds to elements in the Hsp70 gene promoter, resulting in a rapid increase in the expression of Hsp70 [40]. The most intriguing discovery of the present study is that *RIPK1* knockdown further enhanced pMCAO- or OGD-induced decreases in the levels of Hsp90, leading to attenuated Hsp90 binding to Hsf1 in the cytoplasm, promoting the translocation of Hsf1 from the cytoplasm to the nucleus, and in turn, increasing Hsp70.1B mRNA and protein expression. The most abundant heat shock protein, namely, Hsp90, has been characterized as a molecular chaperone that modulates both the structure and function of associated proteins, which are called clients. Numerous kinases and pseudokinases are Hsp90 clients, and these proteins form complexes with Hsp90 and its cochaperone CDC37 [56]. Loss of Hsp90 function likely causes the destabilization and degradation of its clients via the ubiquitin–proteasome pathway. A previous study demonstrated that RIPK1 is an Hsp90 client [57]. Inhibition of Hsp90 function by a specific inhibitor, namely, geldanamycin, disrupts the association between Hsp90 and RIPK1, which results in the degradation of RIPK1 and the subsequent inhibition of TNF-mediated IκB kinase and NF-κB activation [57]. In contrast, we found that ischemia decreased Hsp90, and inhibition of RIPK1 enhanced this ischemia-mediated decrease in the Hsp90 levels in astrocytes. Similarly, Yu et al. reported that treatment with Nec-1 decreased the Hsp90 protein levels during TNF-α-, Smac mimetic- and ZVAD-induced human pulmonary artery endothelial cell necroptosis [31]. In addition, Nec-1 also decreased the binding of Hsp90 to RIPK1/RIPK3/MLKL [30, 31]. Our data and those of others indicate that the interaction of RIPK1 and Hsp90 causes them to stabilize each other, and inhibition of either of these two proteins leads to the instability and degradation of the other protein. However, what is the detailed molecular mechanism underlying this role of RIPK1? Ubiquitination is a posttranslational modification that is related to the function of Hsp90. Blank et al. demonstrated increased Hsp90 ubiquitination after treatment with the photodynamic signal transduction inhibitor hypericin [58]. This increased ubiquitination suppressed Hsp90 chaperone function and increased the degradation of Hsp90-specific clients. Another study by Neckers et al. showed that Swe1-mediated phosphorylation of Hsp90 promotes its ubiquitination and targets Hsp90 for degradation [59]. Based on these previous findings, we reasonably speculate that inhibition of RIPK1 might result in the phosphorylation of Hsp90, promoting its ubiquitination and degradation and leading to decreased Hsp90 levels. This mechanism remains to be investigated in the very near future.

In summary, the significant findings of this current study on ischemic stroke include the following: (1) lysosomal Hsp70.1B, which is regulated by RIPK1, plays a key role in ischemic stroke-induced astrocytic lysosomal membrane damage. (2) Genetic or pharmacological inhibition of RIPK1 inhibits ischemic stroke-induced lysosomal membrane destabilization through the upregulation of Hsp70.1B at the lysosomal membrane in astrocytes. Knockdown of *Hsp70.1B* exacerbates ischemic stroke-mediated changes in LMP and brain injury and prevents the Nec-1-mediated protective effects on lysosomal membranes. (3) The mechanism by which RIPK1 inhibition increases lysosomal Hsp70.1B levels may be associated with an increase in Hsp70.1B transcription; this occurs via *RIPK1* knockdown-mediated exacerbation of ischemic stroke-induced decreases in the cytoplasmic levels of Hsp90 and its interaction with Hsf1, which in turn promotes the nuclear translocation of Hsf1 and increases Hsp70.1B mRNA transcription. One of the limitations of our study is that the shRNA RIPK1 sequence we used was not astrocyte specific; thus, we cannot exclude the possibility that the lentivirus carrying shRNA RIPK1, which was injected i.c.v., also affected microglia and oligodendrocytes in the ischemic zone. Further studies using astrocyte-specific genetic disruption of RIPK1 function will be critical in establishing the mechanisms by which RIPK1 inhibition decreases the Hsp90 levels in ischemic stroke pathology (Fig. 10).

## Supplementary information

Original data

Supplementary tables
