# Supplementary material for: RIPK1 inhibition contributes to lysosomal membrane stabilization in ischemic astrocytes via a lysosomal Hsp70.1B-dependent mechanism
Source: Acta Pharmacol Sin. 2023 Apr 13;44(8):1549–63. doi: 10.1038/s41401-023-01069-8 (PMC10374908; doi:10.1038/s41401-023-01069-8)
Supplement: Supplementary file 1 — Original data [file 41401_2023_1069_MOESM1_ESM.pdf]

Original data

RIPK1 inhibition contributes to lysosomal membrane stabilization in ischemic astrocytes via a lysosomal Hsp70.1B-dependent mechanism

Running head: RIPK1 inhibition stabilizes lysosomal membranes

Hua-Ping Du[2], Yi Guo[1], Yong-Ming Zhu[1], De-fei Gao[1], Bo Lin[1], Yuan Liu[2], Yuan Xu[2], Ali Said[1], Taous Khan[5], Li-Jun Liu[4], Jian-Jun Zhu[4], Yong Ni, [1, 3, *], Hui-Ling Zhang[1, *]

These authors contributed equally: Hua-Ping Du, Yi Guo, Yong-Ming Zhu

All brain slices of TTC scaning in Fig. 5a

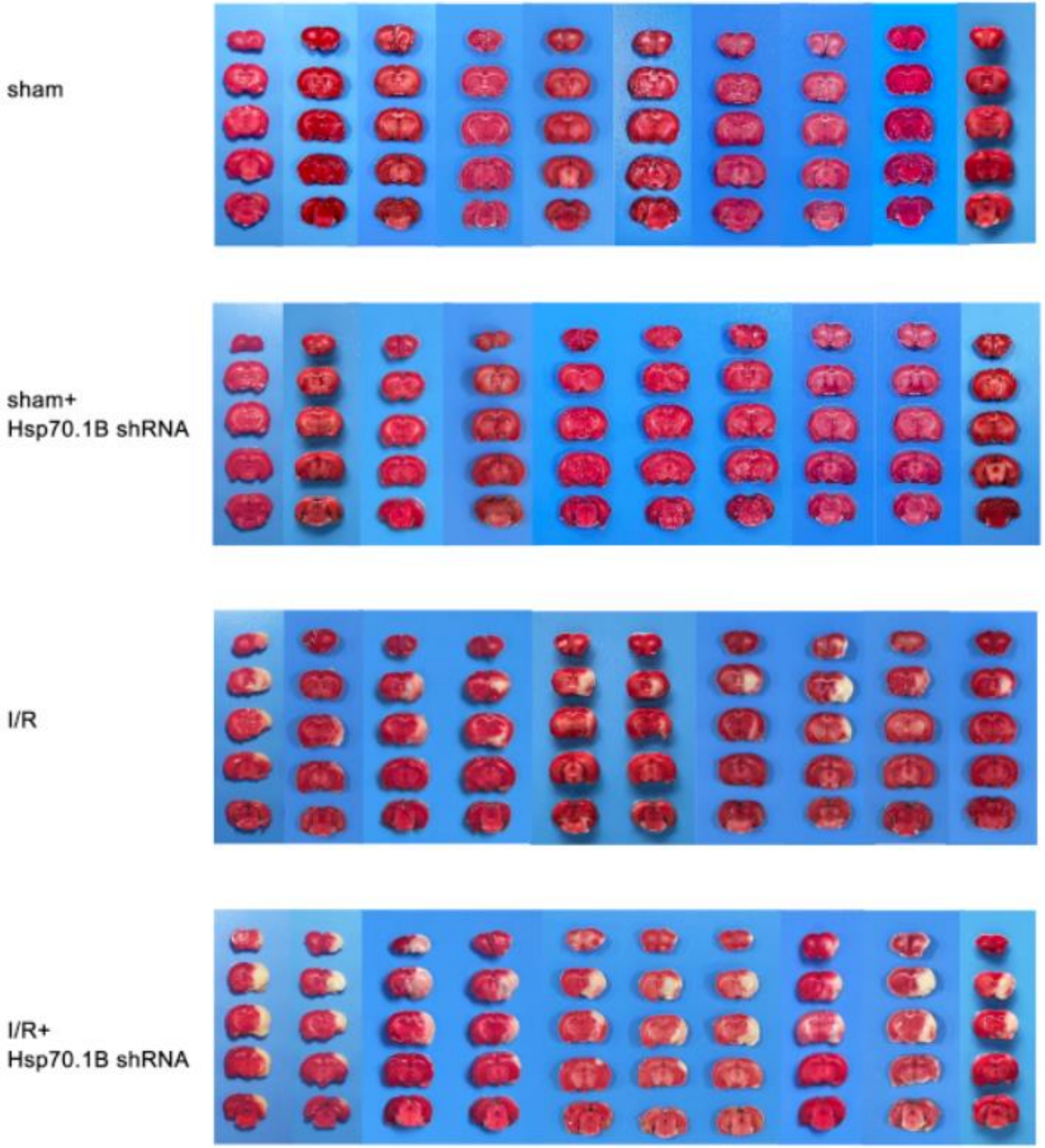
sham
sham+
Hsp70.1B shRNA
I/R
I/R+
Hsp70.1B shRNA

Original data for all Western blotting bands

Fig. 1a

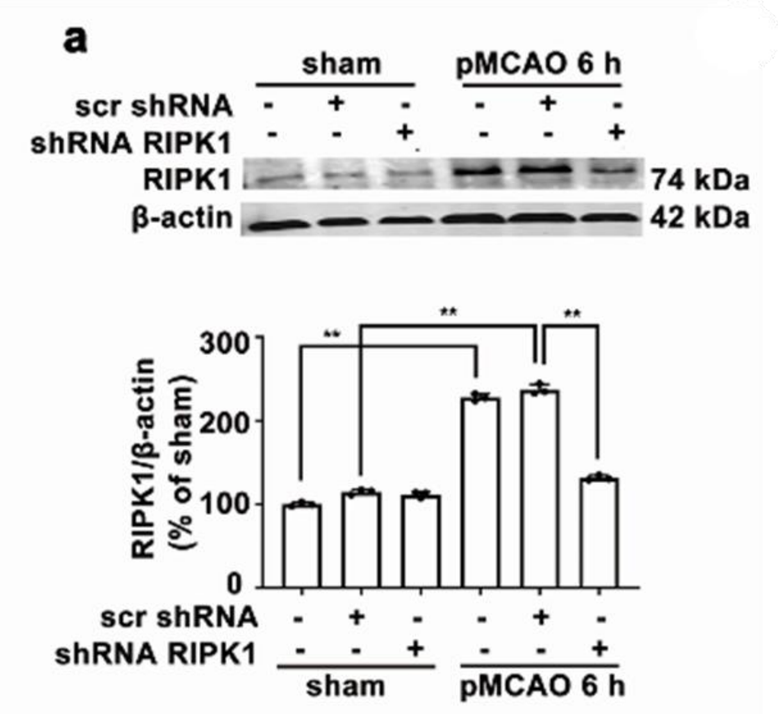
a
sham
pMCAO 6 h
scr shRNA - + - - + -
shRNA RIPK1 - - + - - +
RIPK1
74 kDa
β-actin
42 kDa
RIPK1/β-actin (% of sham)
300
200
100
0
**
**
**
scr shRNA - + - - + -
shRNA RIPK1 - - + - - +
sham
pMCAO 6 h

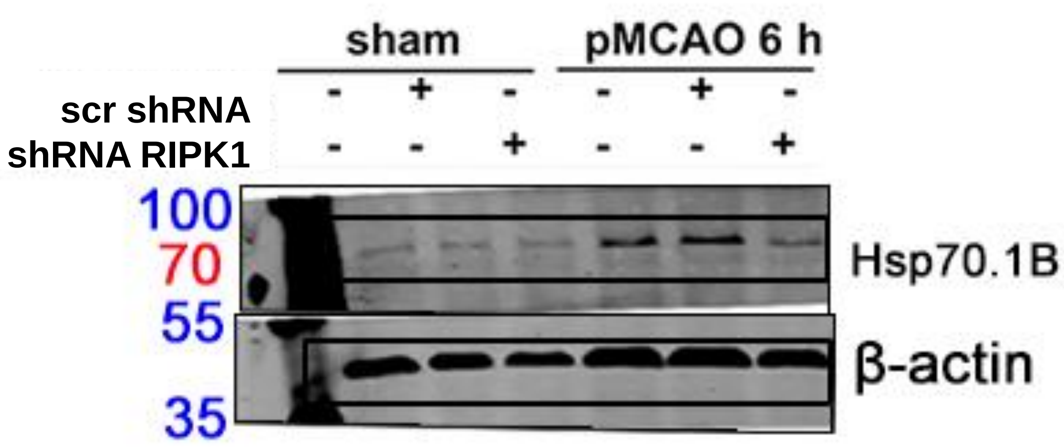
sham
pMCAO 6 h
scr shRNA - + - - + -
shRNA RIPK1 - - + - - +
100
70
55
35
Hsp70.1B
β-actin

Fig. 1c

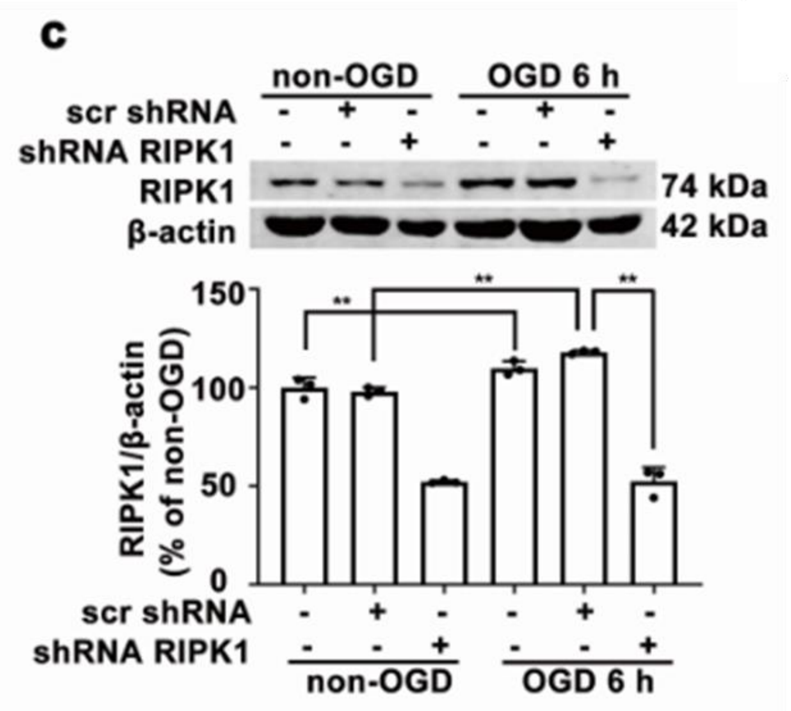
c
non-OGD
OGD 6 h
scr shRNA
shRNA RIPK1
RIPK1
β-actin
74 kDa
42 kDa
RIPK1/β-actin
(% of non-OGD)
150
100
50
0
**
**
**
scr shRNA
shRNA RIPK1
non-OGD
OGD 6 h

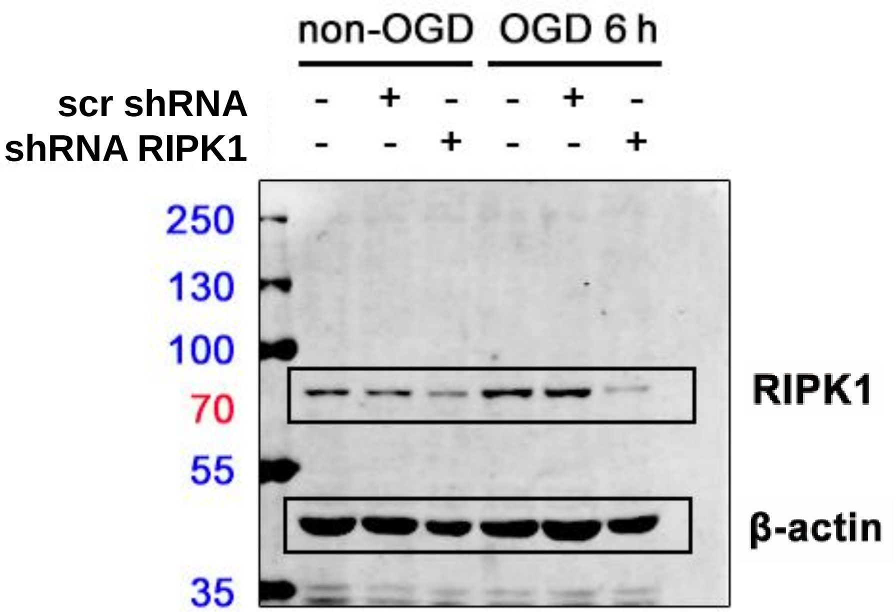
non-OGD
OGD 6 h
scr shRNA
shRNA RIPK1
250
130
100
70
55
35
RIPK1
β-actin

Fig. 2a

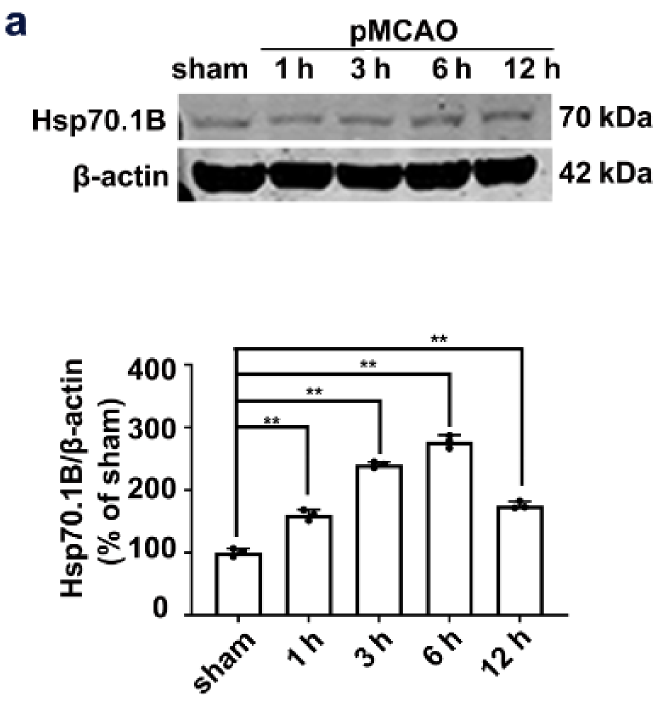
a
pMCAO
sham
1 h
3 h
6 h
12 h
Hsp70.1B
β-actin
70 kDa
42 kDa
Hsp70.1B/β-actin (% of sham)
400
300
200
100
0
**
**
**
**
sham
1 h
3 h
6 h
12 h

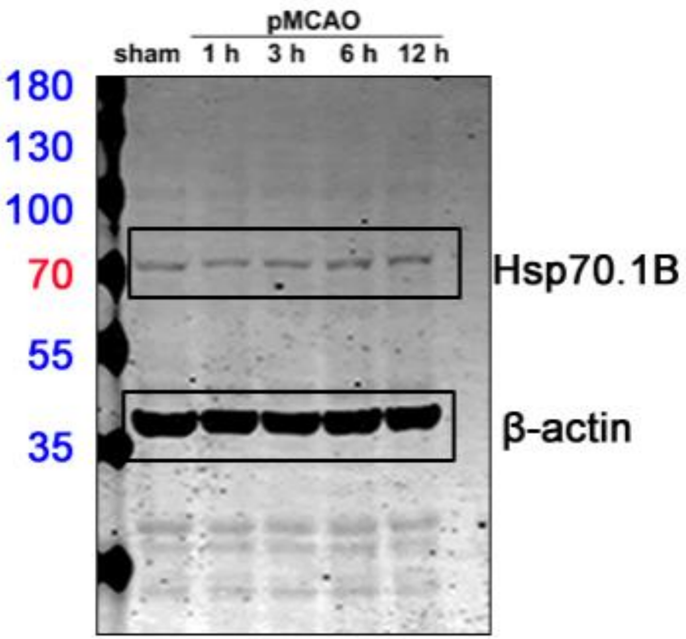
pMCAO
sham
1 h
3 h
6 h
12 h
180
130
100
70
55
35
Hsp70.1B
β-actin

Fig. 2b

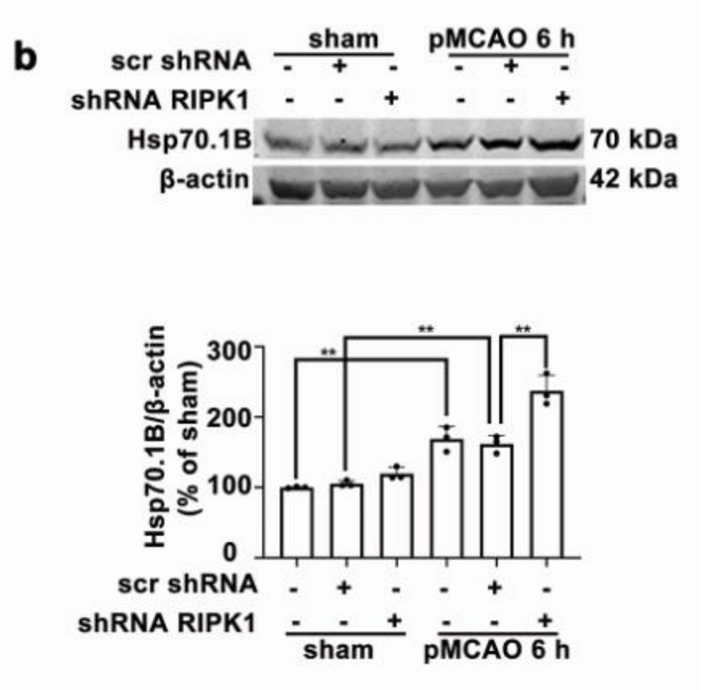
b
sham
pMCAO 6 h
scr shRNA
shRNA RIPK1
Hsp70.1B
β-actin
70 kDa
42 kDa
Hsp70.1B/β-actin (% of sham)
300
200
100
0
**
sham
pMCAO 6 h

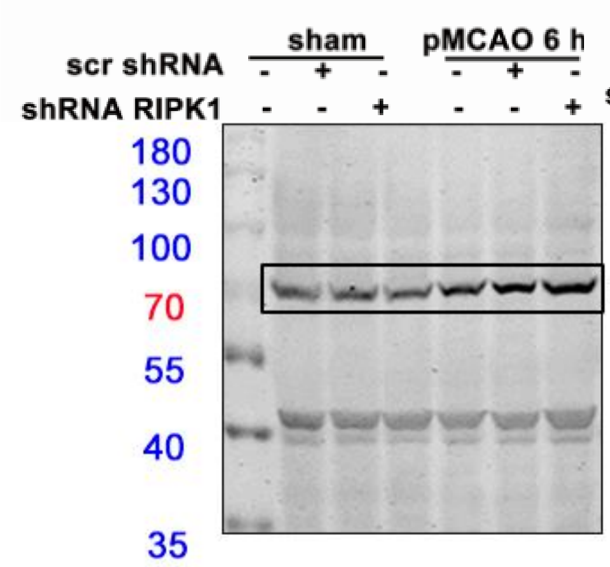
sham
pMCAO 6 h
scr shRNA
shRNA RIPK1
180
130
100
70
55
40
35
Hsp70.1B

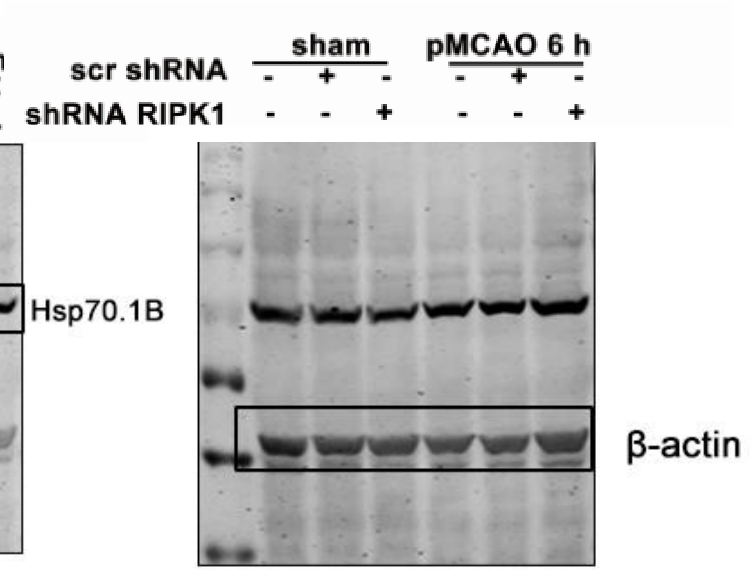
sham
pMCAO 6 h
scr shRNA
shRNA RIPK1
β-actin

Fig. 3a

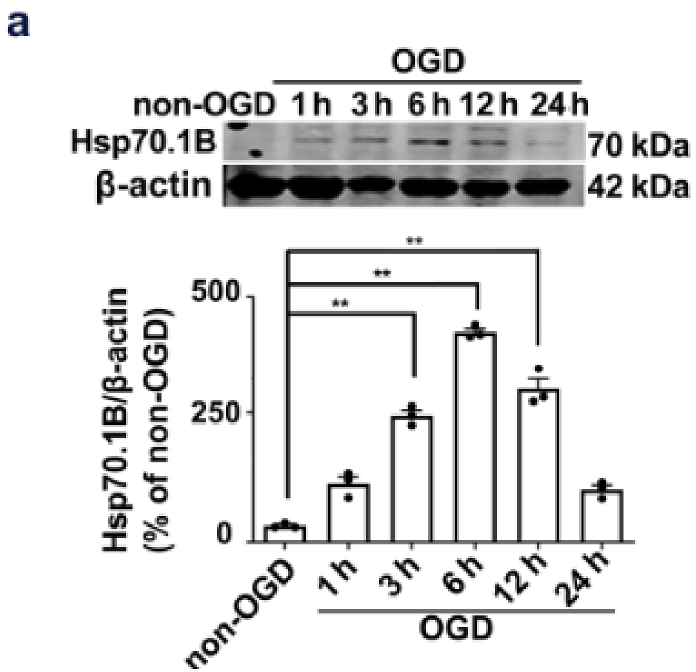
a
OGD
non-OGD 1 h 3 h 6 h 12 h 24 h
Hsp70.1B
70 kDa
β-actin
42 kDa
Hsp70.1B/β-actin
(% of non-OGD)
500
250
0
**
**
**
non-OGD
1 h
3 h
6 h
12 h
24 h
OGD

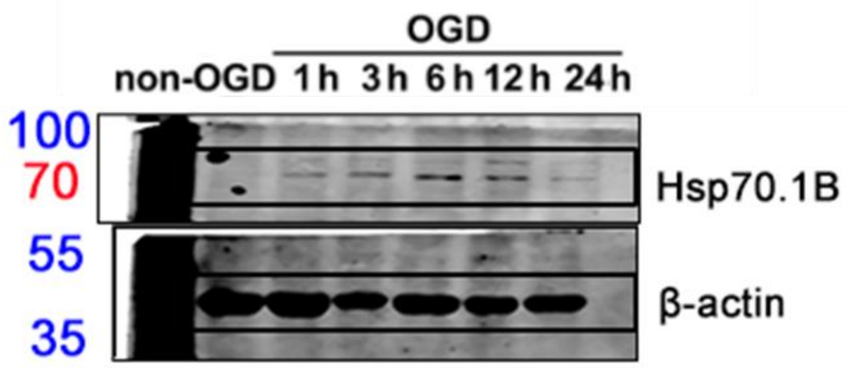
OGD
non-OGD 1 h 3 h 6 h 12 h 24 h
100
70
55
35
Hsp70.1B
β-actin

Fig. 3b

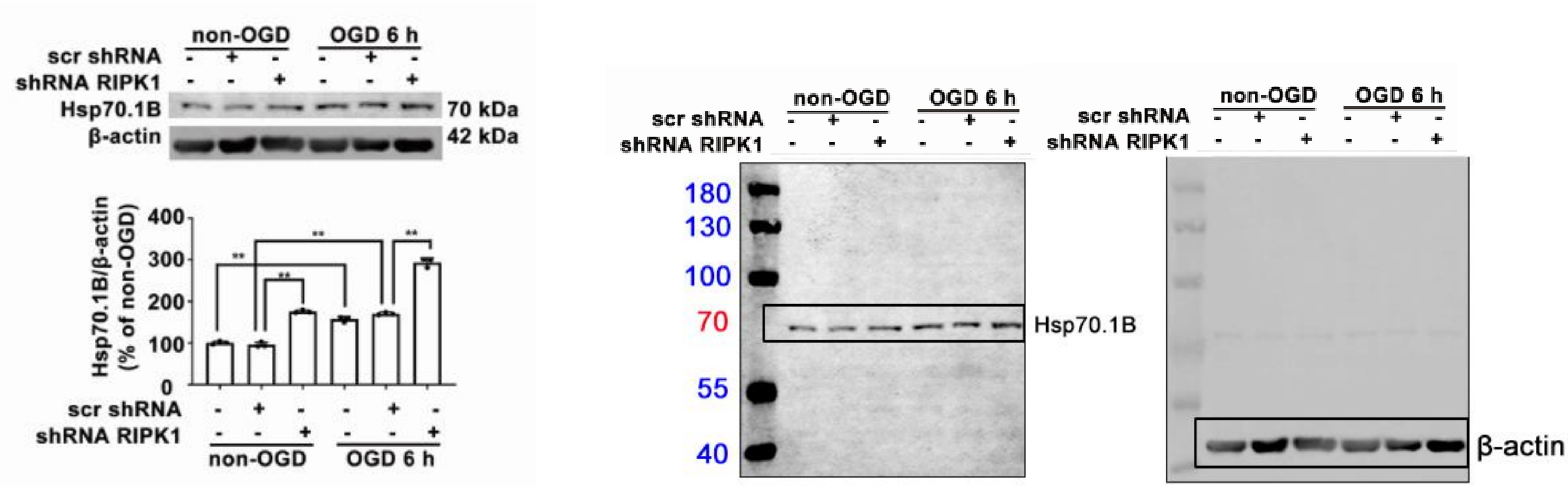
non-OGD
OGD 6 h
scr shRNA - + - - + -
shRNA RIPK1 - - + - - +
Hsp70.1B
70 kDa
β-actin
42 kDa
Hsp70.1B/β-actin (% of non-OGD)
400
300
200
100
0
**
**
**
**
scr shRNA - + - - + -
shRNA RIPK1 - - + - - +
non-OGD
OGD 6 h
non-OGD
OGD 6 h
scr shRNA - + - - + -
shRNA RIPK1 - - + - - +
180
130
100
70
55
40
Hsp70.1B
non-OGD
OGD 6 h
scr shRNA - + - - + -
shRNA RIPK1 - - + - - +
β-actin

Fig. 4a

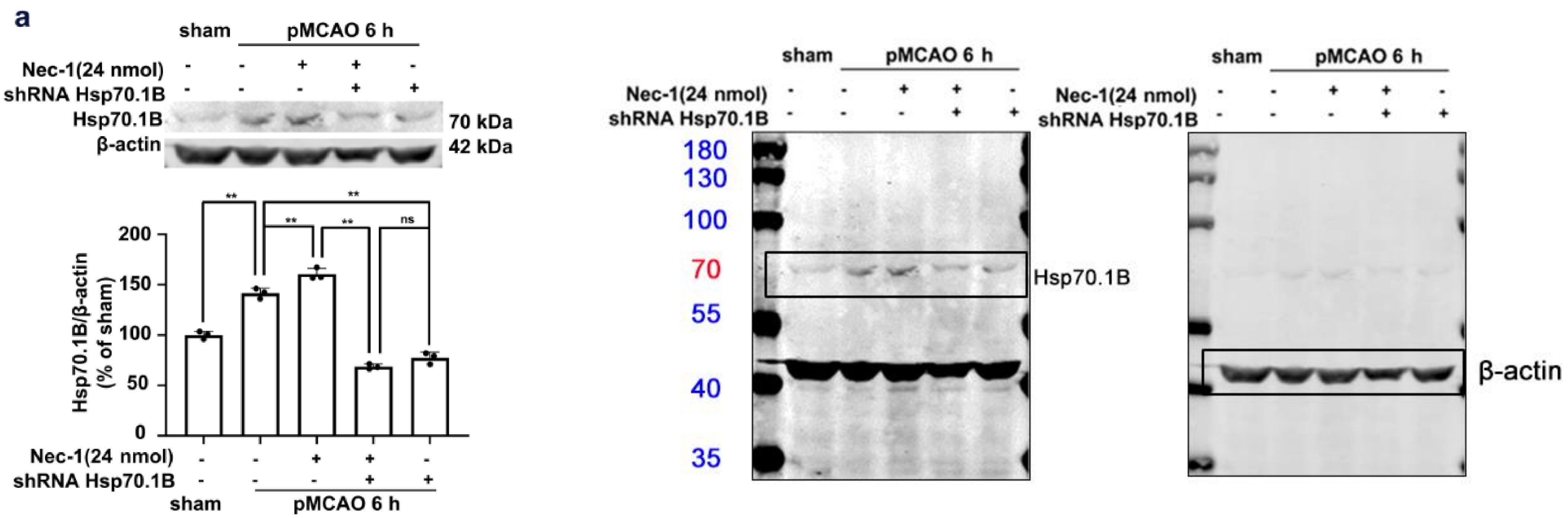
a
sham
pMCAO 6 h
Nec-1(24 nmol)
shRNA Hsp70.1B
Hsp70.1B
β-actin
70 kDa
42 kDa
**
**
**
**
ns
Hsp70.1B/β-actin (% of sham)
200
150
100
50
0
180
130
100
70
55
40
35
Hsp70.1B
β-actin

Fig. 4b

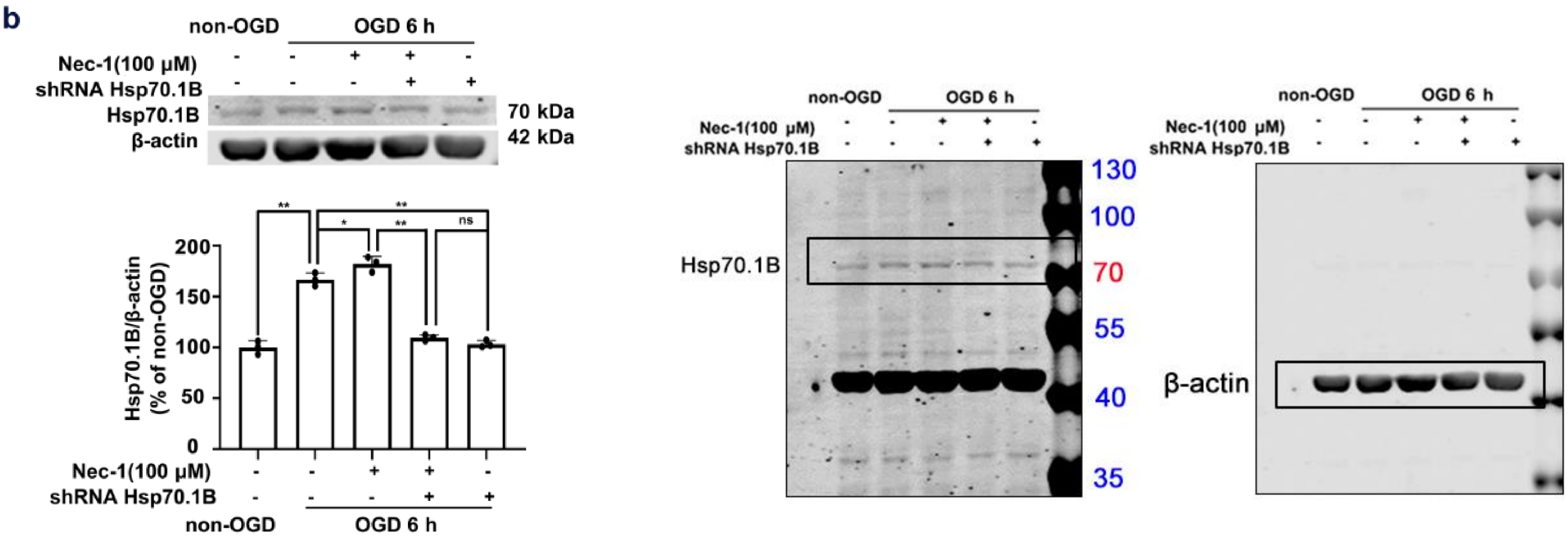
b
non-OGD
OGD 6 h
Nec-1(100 μM)
shRNA Hsp70.1B
Hsp70.1B
β-actin
70 kDa
42 kDa
Hsp70.1B/β-actin (% of non-OGD)
200
150
100
50
0
**
*
ns
130
100
70
55
40
35
Nec-1(100 μM)
shRNA Hsp70.1B
Hsp70.1B
β-actin

Fig. 6a

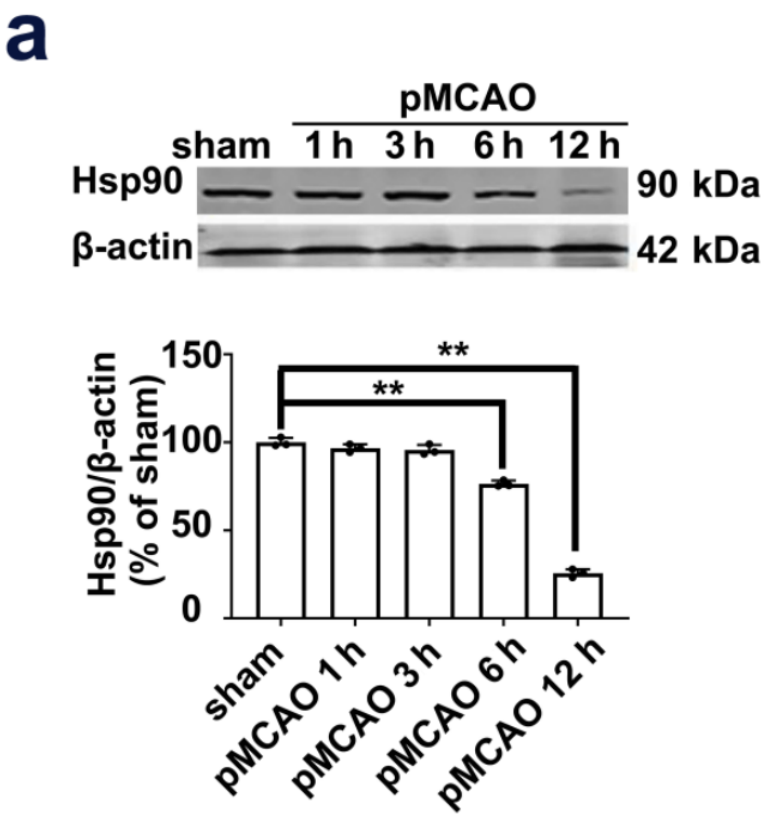

a
pMCAO
sham
1 h
3 h
6 h
12 h
Hsp90
90 kDa
β-actin
42 kDa
Hsp90/β-actin (% of sham)
150
100
50
0
**
**
sham
pMCAO 1 h
pMCAO 3 h
pMCAO 6 h
pMCAO 12 h

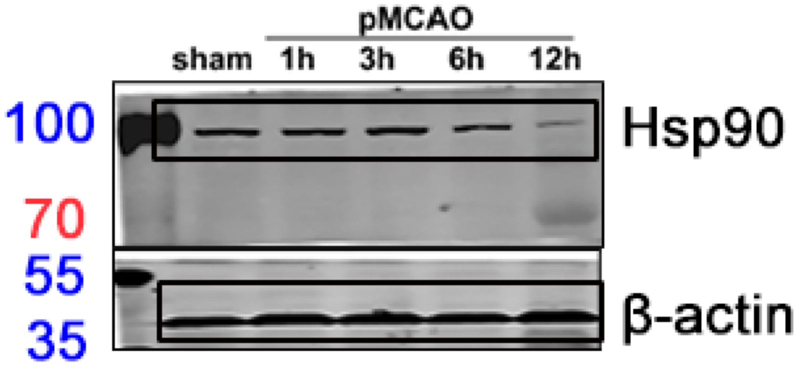

pMCAO
sham
1h
3h
6h
12h
100
70
55
35
Hsp90
β-actin

Fig. 6b

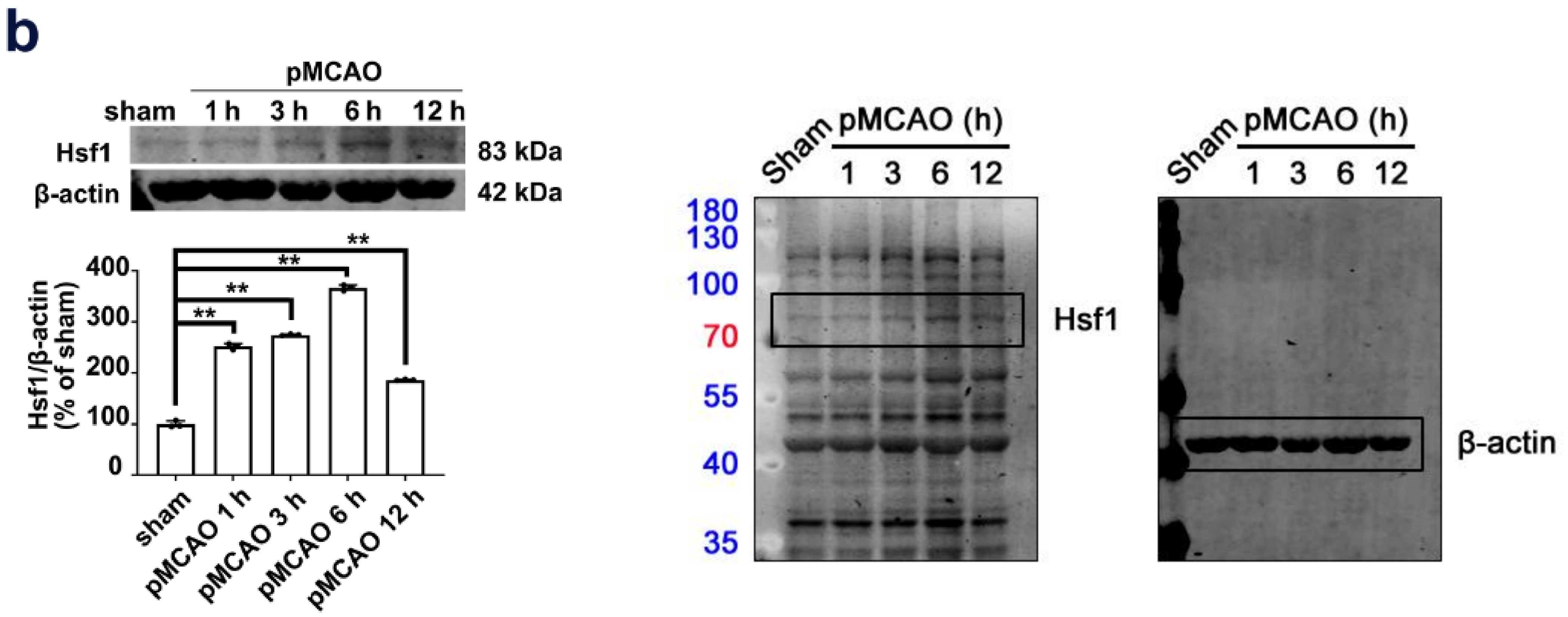
b
pMCAO
sham
1 h
3 h
6 h
12 h
Hsf1
β-actin
83 kDa
42 kDa
Hsf1/β-actin
(% of sham)
400
300
200
100
0
**
**
**
**
sham
pMCAO 1 h
pMCAO 3 h
pMCAO 6 h
pMCAO 12 h
Sham
pMCAO (h)
1
3
6
12
180
130
100
70
55
40
35
Hsf1
Sham
pMCAO (h)
1
3
6
12
β-actin

Fig. 6c

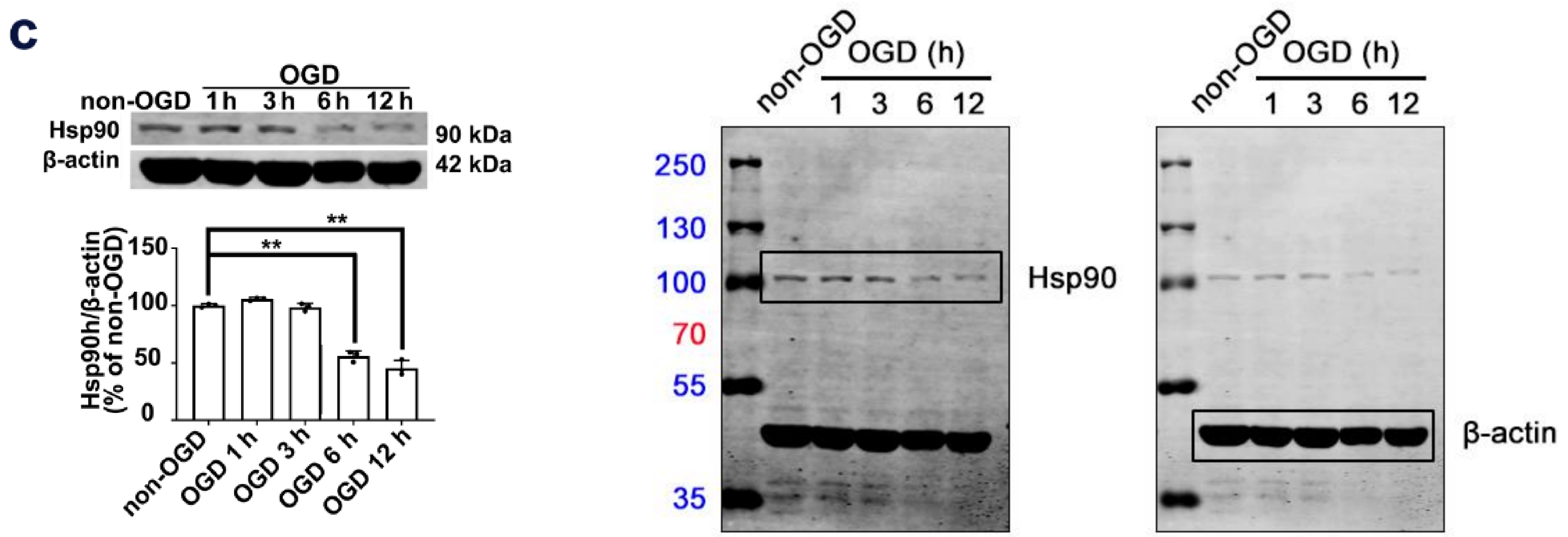
c
OGD
non-OGD 1 h 3 h 6 h 12 h
Hsp90
β-actin
90 kDa
42 kDa
Hsp90h/β-actin (% of non-OGD)
150
100
50
0
**
**
non-OGD
OGD 1 h
OGD 3 h
OGD 6 h
OGD 12 h
non-OGD
OGD (h)
1 3 6 12
250
130
100
70
55
35
Hsp90
non-OGD
OGD (h)
1 3 6 12
β-actin

Fig. 6d

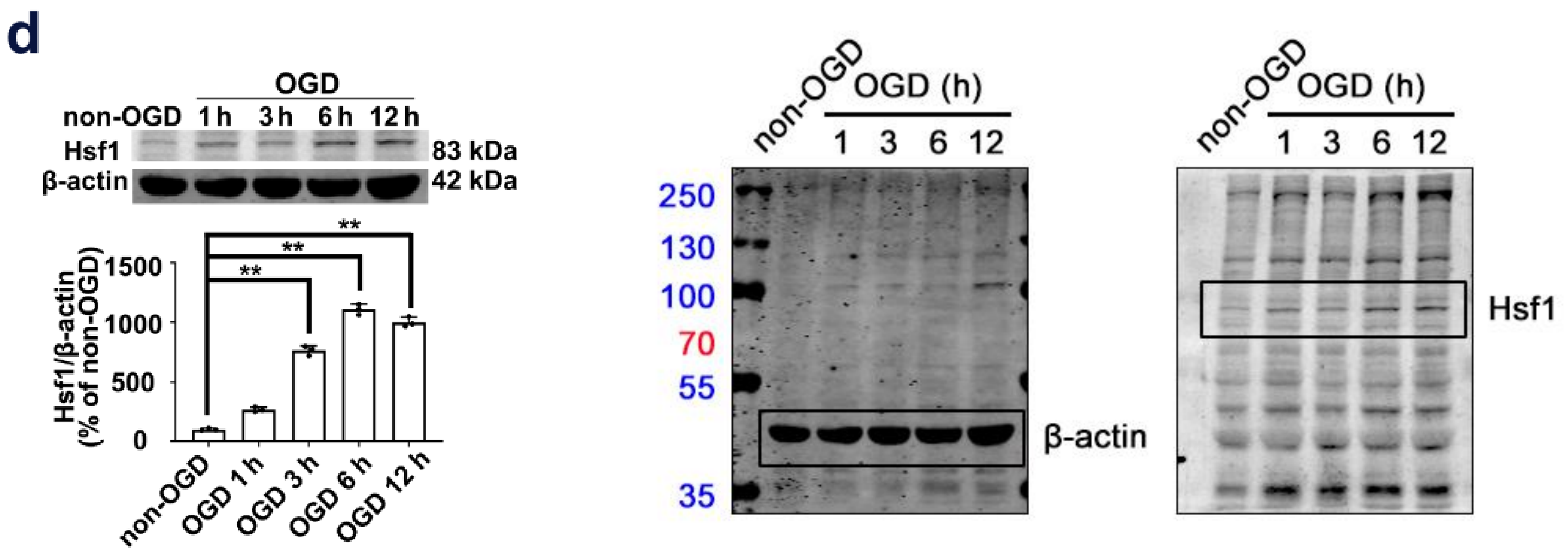
d
OGD
non-OGD 1 h 3 h 6 h 12 h
Hsf1
β-actin
83 kDa
42 kDa
**
**
**
1500
1000
500
0
Hsf1/β-actin
(% of non-OGD)
non-OGD
OGD 1 h
OGD 3 h
OGD 6 h
OGD 12 h
non-OGD
OGD (h)
1 3 6 12
250
130
100
70
55
35
β-actin
non-OGD
OGD (h)
1 3 6 12
Hsf1

Fig. 6e

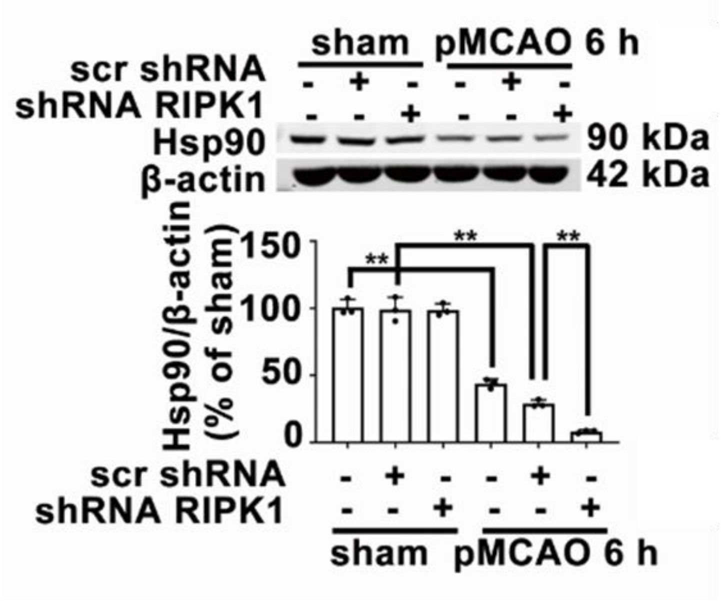
sham
pMCAO 6 h
scr shRNA
shRNA RIPK1
Hsp90
β-actin
90 kDa
42 kDa
Hsp90/β-actin
(% of sham)
150
100
50
0
**
**
**
scr shRNA
shRNA RIPK1
sham
pMCAO 6 h

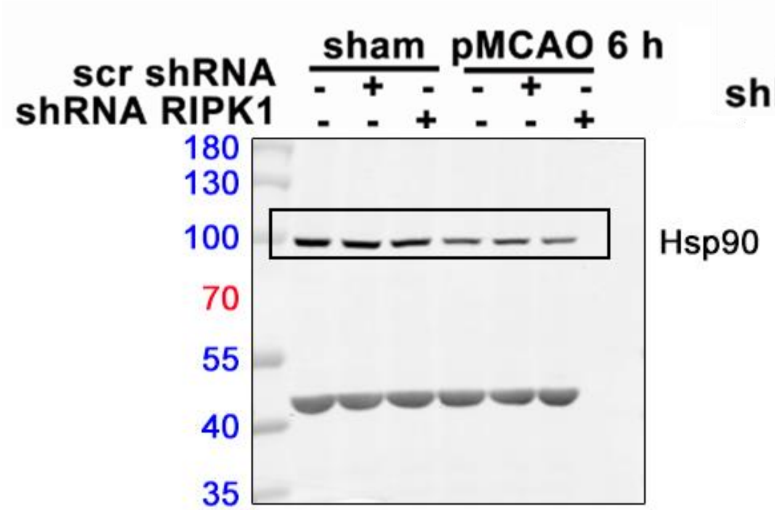
sham
pMCAO 6 h
scr shRNA
shRNA RIPK1
180
130
100
70
55
40
35
Hsp90

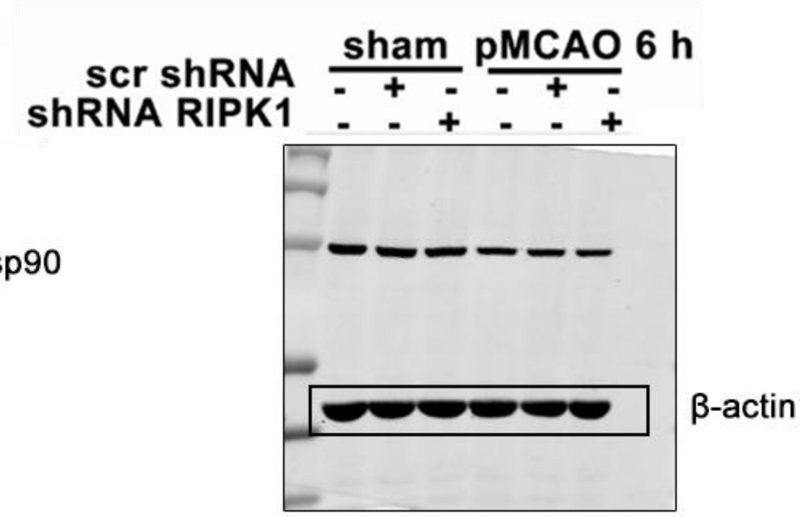
sham
pMCAO 6 h
scr shRNA
shRNA RIPK1
β-actin

Fig. 6f

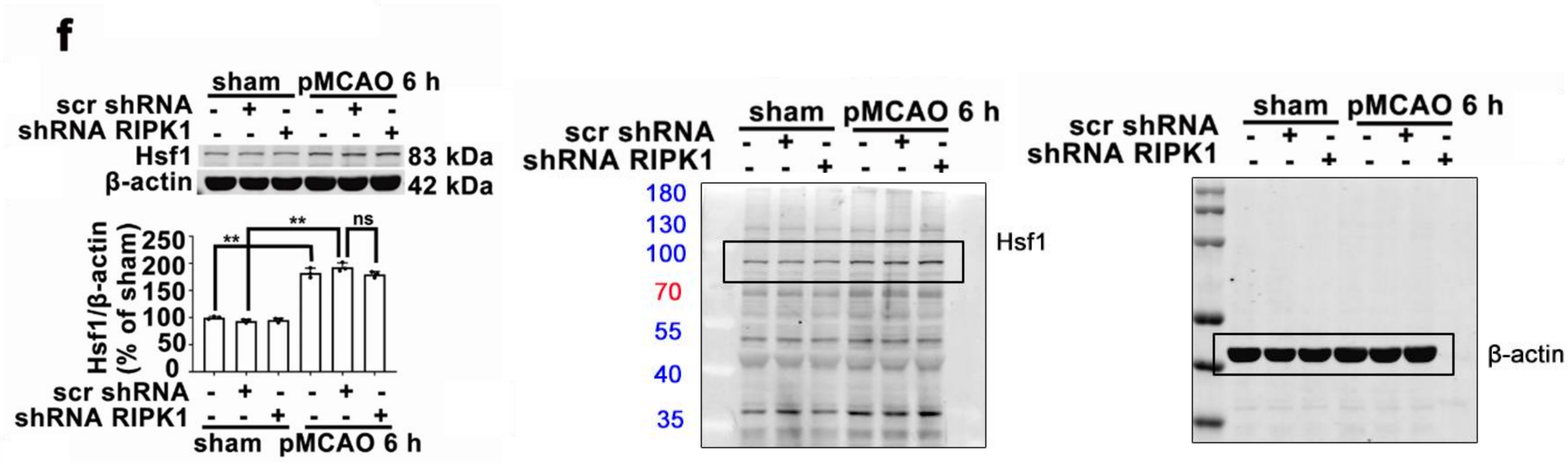
f
sham
pMCAO 6 h
scr shRNA
shRNA RIPK1
Hsf1
β-actin
83 kDa
42 kDa
Hsf1/β-actin (% of sham)
250
200
150
100
50
0
**
**
ns
180
130
100
70
55
40
35
Hsf1
β-actin

Fig. 6g

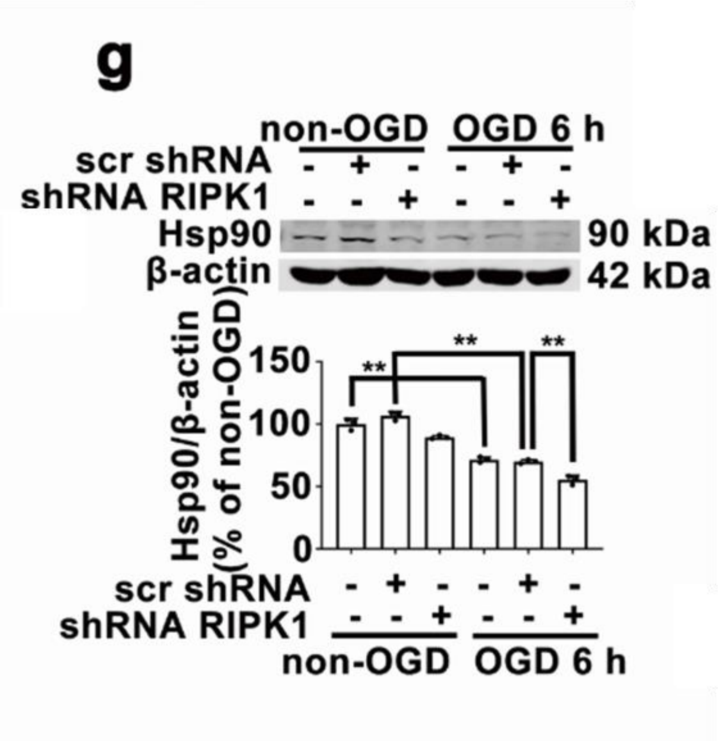

g
non-OGD OGD 6 h
scr shRNA - + - - + -
shRNA RIPK1 - - + - - +
Hsp90 90 kDa
β-actin 42 kDa
Hsp90/β-actin (% of non-OGD)
150
100
50
0
**
**
**
scr shRNA - + - - + -
shRNA RIPK1 - - + - - +
non-OGD OGD 6 h

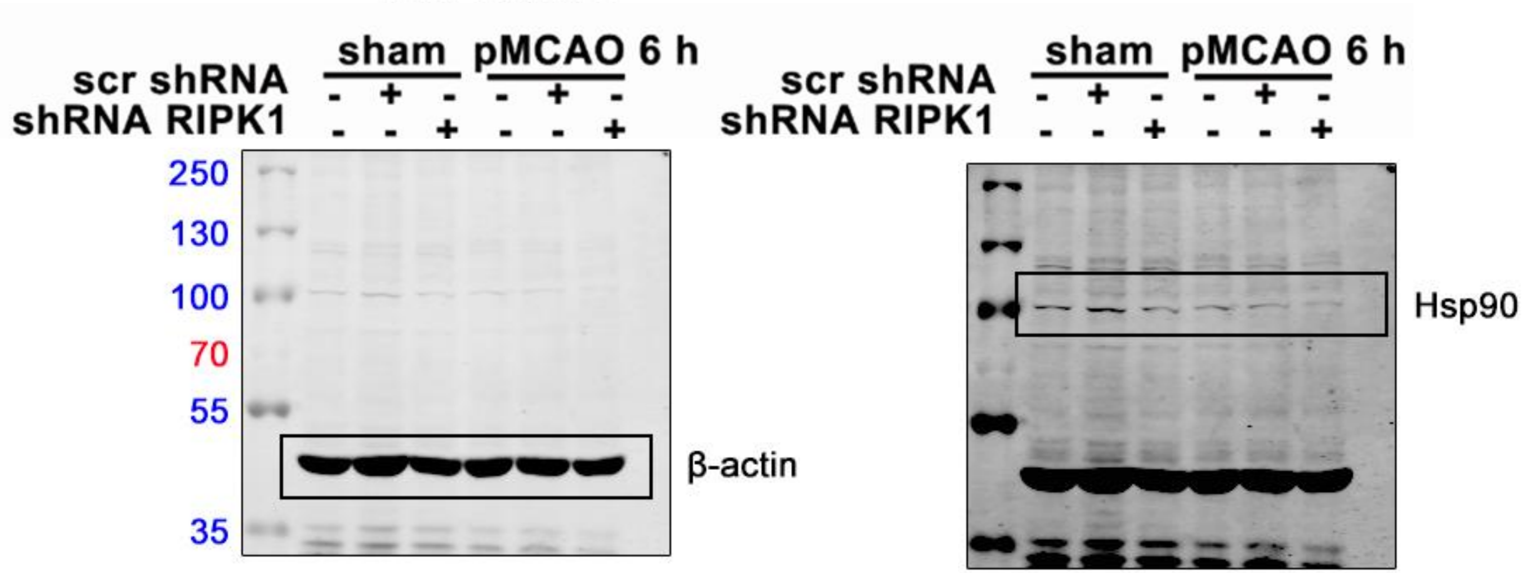

sham pMCAO 6 h
scr shRNA - + - - + -
shRNA RIPK1 - - + - - +
250
130
100
70
55
35
β-actin
sham pMCAO 6 h
scr shRNA - + - - + -
shRNA RIPK1 - - + - - +
Hsp90

Fig. 6h

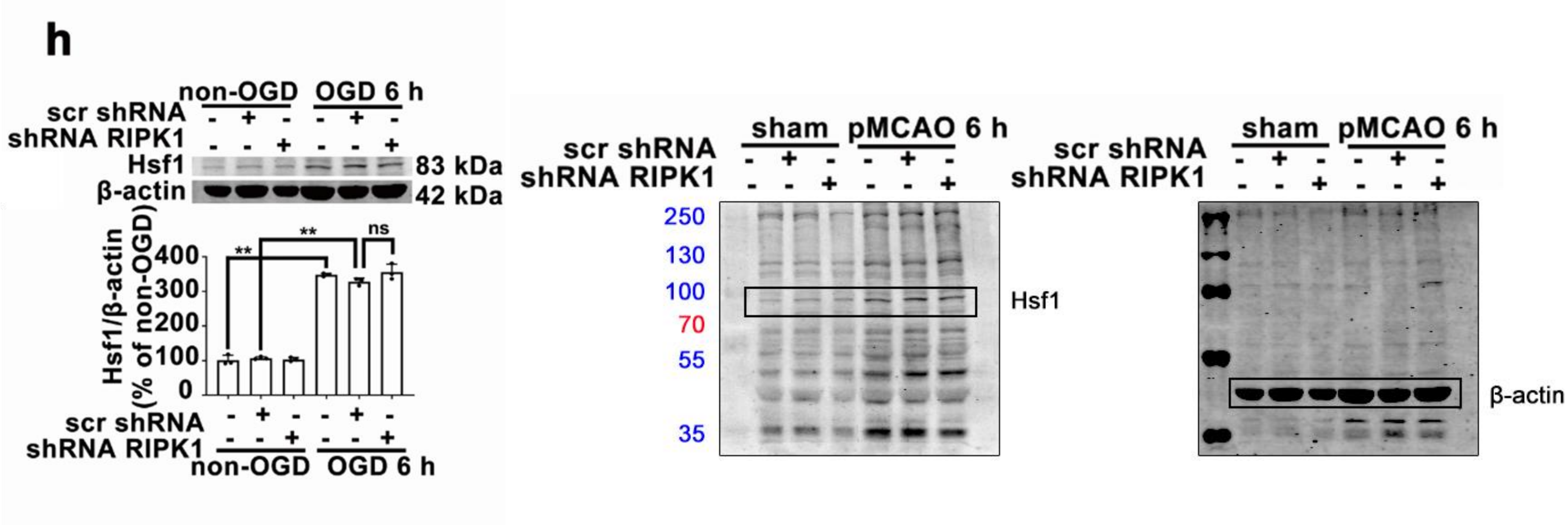
h
non-OGD
OGD 6 h
scr shRNA
shRNA RIPK1
Hsf1
β-actin
83 kDa
42 kDa
Hsf1/β-actin (% of non-OGD)
400
300
200
100
0
**
**
ns
sham
pMCAO 6 h
250
130
100
70
55
35
Hsf1
β-actin

Fig.7a

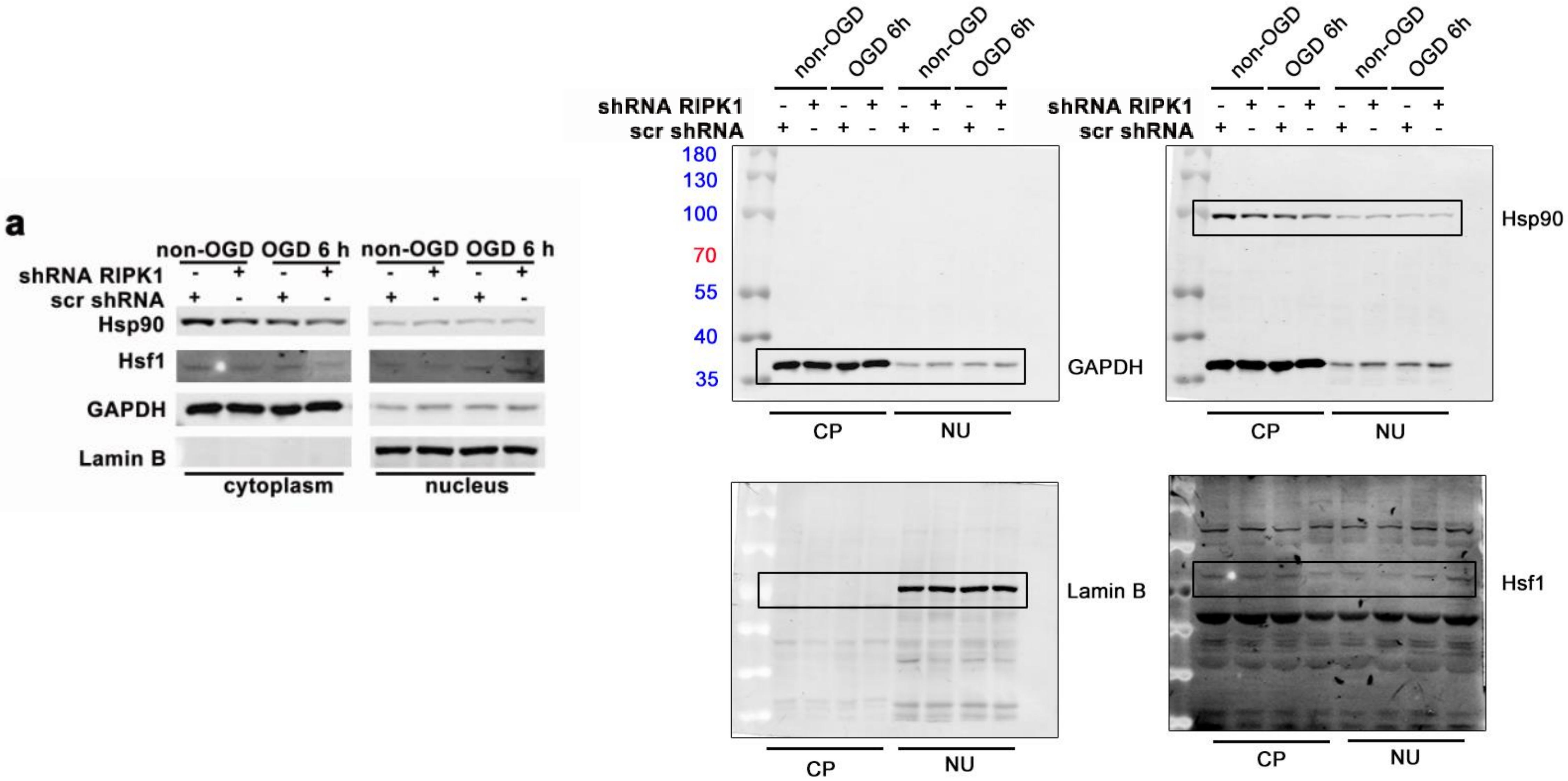
a
non-OGD OGD 6 h non-OGD OGD 6 h
shRNA RIPK1
scr shRNA
Hsp90
Hsf1
GAPDH
Lamin B
cytoplasm
nucleus
non-OGD
OGD 6h
180
130
100
70
55
40
35
GAPDH
Hsp90
Lamin B
Hsf1
CP
NU

Fig.7b

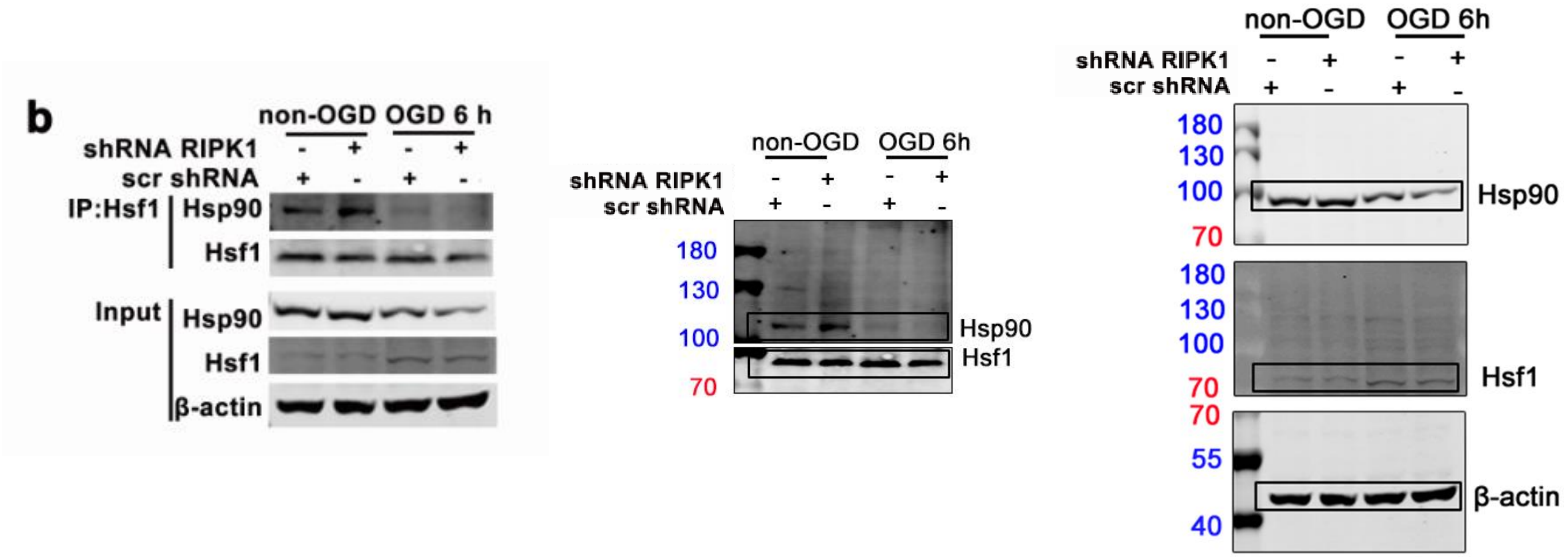
b
non-OGD
OGD 6 h
shRNA RIPK1
scr shRNA
IP:Hsf1
Hsp90
Hsf1
Input
Hsp90
Hsf1
β-actin
non-OGD
OGD 6h
shRNA RIPK1
scr shRNA
180
130
100
70
Hsp90
Hsf1
non-OGD
OGD 6h
shRNA RIPK1
scr shRNA
180
130
100
70
Hsp90
180
130
100
70
Hsf1
70
55
40
β-actin
