# Supplementary material for: RIPK1 inhibition contributes to lysosomal membrane stabilization in ischemic astrocytes via a lysosomal Hsp70.1B-dependent mechanism
Source: Acta Pharmacol Sin. 2023 Apr 13;44(8):1549–63. doi: 10.1038/s41401-023-01069-8 (PMC10374908; doi:10.1038/s41401-023-01069-8)
Supplement: Supplementary file 2 — Supplementary tables [file 41401_2023_1069_MOESM2_ESM.docx]

**Supplementary tables**

**Supplementary Table 1. Primary antibodies used in this study**

| **Protein** | **Usage** | **Antibody** |
| --- | --- | --- |
| GFAP | IHC (1:500) | C9205, Sigma |
| GFAP | IHC (1:500) | AB5804, Millipore |
| Lamp1 | IHC (1:200), IF (1:200) | ab24170, Abcam |
| Lamp1 | IHC (1:200), IF (1:200), WB (1:300), | ab13523, Abcam |
| Hsp70.1B | IHC (1:200), IF (1:300), WB (1:1000) | GTX106148, Gene Tex |
| Hsf1 | IHC (1:100), IF (1:200), WB (1:500) | ab2923, Abcam |
| Hsp90 | IHC (1:100), IF (1:200), WB (1:500) | ab13492, Abcam |
| RIPK1 | WB (1:500) | 610458, BD |
| β-actin | WB (1:5000) | Sigma, A5441 |
| GAPDH | WB (1:1000) | YM3040, Immunoway |
| Lamin B1 | WB (1:1000) | 12987-1-AP, Proteintech |

Abbreviations: WB, Western blotting; IF, Immunofluorescence; IHC,Immunohistochemistry

**Supplementary Table 2. Secondary antibodies used in this study**

| **Protein** | **Usage** | **Antibody** |
| --- | --- | --- |
| Alexa Fluor® 594 goat anti-rabbit IgG | IF (1:500),IHC(1:500) | A11012, lifetechnologies |
| Alexa Fluor® 594 goat anti-mouse IgG | IF (1:500),IHC(1:500) | A11005, lifetechnologies |
| Alexa Fluor® 488 goat anti-rabbit IgG | IF (1:500),IHC(1:500) | A11008, lifetechnologies |
| Alexa Fluor® 488 goat anti-mouse IgG | IF (1:500),IHC(1:500) | A11001, lifetechnologies |
| Anti-mouse IgG | WB (1:10000) | 042-06-18-06, KPL |
| anti-rabbit IgG | WB (1:10000) | 042-06-15-06, KPL |

Abbreviations: WB, Western blotting; IF, Immunofluorescence, IHC, Immunohistochemistry
